# An early ABA-induced stomatal closure, Na^+^ sequestration in leaf vein and K^+^ retention in mesophyll confer salt tissue tolerance in *Cucurbita* species

**DOI:** 10.1093/jxb/ery251

**Published:** 2018-07-10

**Authors:** Mengliang Niu, Junjun Xie, Chen Chen, Haishun Cao, Jingyu Sun, Qiusheng Kong, Sergey Shabala, Lana Shabala, Yuan Huang, Zhilong Bie

**Affiliations:** 1 College of Horticulture and Forestry Sciences, Huazhong Agricultural University/Key Laboratory of Horticultural Plant Biology, Ministry of Education, Wuhan, P. R. China; 2 Department of Horticulture, Foshan University, Foshan, P. R. China; 3 Tasmanian Institute for Agriculture, College of Science and Engineering, University of Tasmania, Hobart, Tasmania, Australia

**Keywords:** ABA, *HKT1*, K^+^ retention, leaf vein, Na^+^ sequestration, stomatal closure

## Abstract

Tissue tolerance to salinity stress is a complex physiological trait composed of multiple ‘sub-traits’ such as Na^+^ compartmentalization, K^+^ retention, and osmotic tolerance. Previous studies have shown that some *Cucurbita* species employ tissue tolerance to combat salinity and we aimed to identify the physiological and molecular mechanisms involved. Five *C. maxima* (salt-tolerant) and five *C. moschata* (salt-sensitive) genotypes were comprehensively assessed for their salt tolerance mechanisms and the results showed that tissue-specific transport characteristics enabled the more tolerant lines to deal with the salt load. This mechanism was associated with the ability of the tolerant species to accumulate more Na^+^ in the leaf vein and to retain more K^+^ in the leaf mesophyll. In addition, *C. maxima* more efficiently retained K^+^ in the roots when exposed to transient NaCl stress and it was also able to store more Na^+^ in the xylem parenchyma and cortex in the leaf vein. Compared with *C. moschata*, *C. maxima* was also able to rapidly close stomata at early stages of salt stress, thus avoiding water loss; this difference was attributed to higher accumulation of ABA in the leaf. Transcriptome and qRT-PCR analyses revealed critical roles of high-affinity potassium (HKT1) and intracellular Na^+^/H^+^ (NHX4/6) transporters as components of the mechanism enabling Na^+^ exclusion from the leaf mesophyll and Na^+^ sequestration in the leaf vein. Also essential was a higher expression of *NCED3*s (encoding 9-cis–epoxycarotenoid dioxygenase, a key rate-limiting enzyme in ABA biosynthesis), which resulted in greater ABA accumulation in the mesophyll and earlier stomata closure in *C. maxima*.

## Teaser

Tissue tolerance to salinity in *Cucurbita* is associated with HKT1-mediated Na^+^ exclusion from the leaf mesophyll, NHX4/6-mediated Na^+^ sequestration in the leaf vein, K^+^ retention in the leaf mesophyll, and early ABA-induced stomatal closure.

## Introduction

Salinity affects more than 80 million hectares of arable land worldwide, with an estimated annual cost in lost crop production ranging between US$11 and $27 billion (Munns and Tester, 2008; Ismail *et al.*, 2014; Qadir *et al.*, 2014). Improving salt tolerance in major cultivated crops is of paramount importance to global food security in the 21st century, but the process is significantly handicapped by the complexity of salinity tolerance mechanisms (Chakraborty *et al.*, 2016*a*).

To date, most experimental work on salinity tolerance has focused on shoot Na^+^ exclusion (Møller *et al.*, 2009; Huang *et al.*, 2013; Niu *et al.*, 2018). However, there is increasing evidence that this is not the sole mechanism for salinity tolerance (Rajendran *et al.*, 2009; Munns *et al.*, 2016; Zaki and Yokoi, 2016). Some species or genotypes can maintain a relatively high Na^+^ concentration in their shoots without major negative impacts on growth and yield. This strategy, termed a tissue-tolerance mechanism (Munns *et al.*, 2016), is observed in both halophytes (Wang *et al.*, 2007; Adolf *et al.*, 2013) and glycophytes (e.g. *Triticum aestivum*, Genc *et al.*, 2007; *T. monococcum*, Rajendran *et al.*, 2009; lettuce, Bartha *et al.*, 2015; tomato, Gálvez *et al.*, 2012; Zaki and Yokoi, 2016; rice, Prusty *et al.*, 2018).

Tissue tolerance is a complex physiological trait composed of multiple ‘sub-traits’ (Munns *et al.*, 2016), which include Na^+^ compartmentalization (James *et al.*, 2006), K^+^ retention (Wu *et al.*, 2015; Percey *et al.*, 2016), and osmotic tolerance (Munns *et al.*, 2016).

For Na^+^ compartmentalization, Na^+^ is partitioned either within specialized cells (e.g. leaf epidermis and epidermal balder cells, Shabala *et al.*, 2014), or is sequestered in the vacuoles in the leaf mesophyll, so that the cytosolic Na^+^ levels in the functional cells are kept low (Shapira *et al.*, 2009; Munns and Gilliham, 2015). In the latter process, the subcellular Na^+^ compartmentalization is achieved by Na^+^/H^+^ antiporters (NHX) (Bassil *et al.*, 2011; Bassil and Blumwald, 2014). Intracellular NHX proteins are also involved in K^+^ homeostasis; for example, NHX1 and NHX2 are essential for active K^+^ transport into the vacuole (Barragán *et al.*, 2012).

Another aspect of tissue tolerance is K^+^ retention. Enhanced K^+^ retention and the ability of a cell to maintain cytosolic K^+^ homeostasis correlate with salinity tolerance in a broad range of plant species (Yang *et al.*, 2013; Wu *et al.*, 2015; Chakraborty *et al.*, 2016*b*; Percey *et al.*, 2016; Shabala *et al.*, 2016; Shabala, 2017) and are essential for preventing salinity-induced programmed cell death (Shabala, 2009; Demidchik *et al.*, 2010). High cytosolic K^+^ levels are also essential to maintain high vacuolar H^+^-PPase activity, thus enabling the operation of tonoplast NHX proteins that mediate vacuolar Na^+^ sequestration (Shabala, 2003). K^+^ is also the major inorganic osmolyte for tissue osmotic adjustment under stress conditions (Shabala and Lew, 2002).

The third component of the tissue-tolerance mechanism is an osmotic tolerance (Munns *et al.*, 2016; Álvarez-Aragón and Rodríguez-Navarro, 2017). In plants possessing tissue tolerance, accumulated Na^+^ can be used as a low-energy-cost osmolyte for the adjustment of cell turgor and ultimately of tissue growth under salt stress (Munns and Gilliham, 2015). In addition, the immediate constraint imposed by salinity is an osmotic stress. Plant responses to osmotic stress can occur immediately after the roots are exposed to salt, with the rate of shoot growth falling significantly (Munns and Tester, 2008). The most dramatic and readily measurable whole-plant response to salinity is a decrease in the stomatal conductance, which is highly related to osmotic stress (Munns and Tester, 2008). The short-term stomatal responses can reliably identify differences in osmotic stress tolerance in different varieties of durum wheat (Rahnama *et al.*, 2010). Because stomatal conductance is largely controlled by abscisic acid (ABA), changes in its concentration are often used to explain altered stomatal behavior and thus water loss in the leaf (Verslues *et al.*, 2006). Maintaining water content is very important in tolerating high Na^+^ concentrations without suffering toxicity. Biochemical and genetic studies have suggested that 9-cis-epoxycarotenoid dioxygenase (NCED) is the key enzyme in the ABA biosynthetic pathway in plants (Speirs *et al.*, 2013).

With the application of molecular techniques, several key genes mediating Na^+^ and K^+^ transport have been identified. These include the genes encoding the Na^+^/H^+^ antiporter (SOS1, Shi *et al.*, 2002), the high-affinity Na^+^/K^+^-permeable transporter (HKT, Horie *et al.*, 2009), the K^+^ efflux antiporter (KEA, Zheng *et al.*, 2013), the K^+^ transporters (KT/HAK/KUP, Santa-María *et al.*, 2018), and the Na^+^:K^+^:Cl^–^ co-transporter (CCC, Colmenero-Flores *et al.*, 2007). SOS1 has been identified as a Na^+^/H^+^ antiporter localized in epidermal cells in the root apex, where it actively extrudes Na^*+*^ from the cytosol into the rhizosphere (Shi *et al.*, 2002). SOS1 also affects the partitioning of Na^+^ between plant organs (Olías *et al.*, 2009). HKT transporters can retrieve Na^+^ from the xylem and contribute to Na^+^ exclusion from leaves when expressed in the xylem parenchyma cells (Zhang *et al.*, 2018).

Many *Cucurbita* species are vegetable crops that are cultivated and consumed all over the world. The annual production of all *Cucurbita* species reached 32.5 million tons in 2014 (http://www.fao.org/faostat/en/#data/QC). *Cucurbita maxima* and *C. moschata* are two of the three most economically important cultivated species (Sun *et al.*, 2017), and their economic values are increasing as they are also used as rootstocks for other cucurbit crops, including cucumber, watermelon, and melon, to enhance tolerance to soil-borne diseases and abiotic stresses (Kong *et al.*, 2014; Sun *et al.*, 2017). However, our understanding of salt-tolerance mechanisms in *Cucurbita* crops remains rather poor, with most studies focusing on shoot Na^+^ exclusion mechanisms when they are used as rootstocks (Edelstein *et al.*, 2011; Huang *et al.*, 2013; Lei *et al.*, 2014; Niu *et al.*, 2018). Our previous studies have shown that some *C. maxima* genotypes employ tissue tolerance to combat salinity, in contrast with *C. moschata* (Xie *et al.*, 2015; Niu *et al.*, 2017); however, the specific mechanism behind this remains elusive.

The aim of this work was to investigate the contribution of physiological and molecular mechanisms towards tissue tolerance to salinity in *Cucurbita*. This was done using a range of physiological (ion, ABA, gas exchange analysis), electrophysiological (the MIFE technique for non-invasive microelectrode ion flux measurements), microscopy (energy-dispersive X-ray microanalysis, SEM-EDX), and molecular (transcriptome, qRT-PCR) techniques. Our results suggest that the overall tissue tolerance to salinity in *Cucurbita* requires three concurrently operating mechanisms: (1) *HKT1*-mediated Na^+^ exclusion from the leaf mesophyll and *NHX4/6*-mediated Na^+^ sequestration in the leaf vein; (2) K^+^ retention in the leaf mesophyll; and (3) early ABA-induced stomatal closure.

## Materials and methods

### Plant material and experimental protocols

Five *Cucurbita maxima* (R660, R655, R664, R686, and N12) and five *C. moschata* (L24, L13, N15, L6, and L14) genotypes contrasting in their salinity tolerance were grown under substrate culture (peat:perlite 2:1, v/v). All the genotypes are inbred lines constructed by our group. Plants were cultivated and salt treatments were applied as described by Niu *et al.* (2017). Briefly, plants at the three true-leaf stage were treated with 150 mM NaCl and were harvested at day 15 of the salt treatment. Plant total dry weight and Na^+^ and K^+^ contents of the leaf mesophyll and all the leaf veins were measured, and the salt tolerance of each genotype was determined as the relative plant dry weight (dry weight under NaCl treatment divided by dry weight under control conditions, expressed as a percentage).

For physiological assessment of the effects of salinity, plants were cultivated in hydroponics as described by Xie *et al.* (2015). At the three true-leaf stage, plants were treated with 100 mM NaCl. Leaf relative water content (RWC), ABA content, and gas exchange parameters were measured, and X-ray microanalysis of Na^+^ in the xylem parenchyma and cortex in leaf vein transverse sections were conducted.

To evaluate the contribution of osmotic effects to the salt tolerance, representative genotypes of *C. maxima* and *C. moschata* (N12 and N15, respectively) were grown hydroponically as described by Xie *et al.* (2015). At the three true-leaf stage, plants were treated with either 100 mM NaCl or isotonic 170 mM sorbitol, while unstressed plants were used as controls. Leaf stomatal conductance (*g*_s_), transpiration rate (*T*_r_), net photosynthetic rate (*P*_n_), and RWC were measured after 4 h of treatment.

### Non-invasive measurements of ion flux

*Cucurbita* seeds were surface-sterilized with 1% HClO for 30 min and rinsed thoroughly with distilled water, and then incubated in the dark at 26 °C until germination. The germinated seeds were then directly grown hydroponically in aerated basic salt media (BSM) solution (0.5 mM KCl, 0.1 mM CaCl_2_, pH 5.7 non-buffered) in the dark at room temperature (24 °C). Six-day-old seedlings with primary roots 50–60 mm long were used for the experiments. For leaf mesophyll and leaf vein (primary vein) measurements, the plants were grown in 0.5-l plastic pots filled with peat moss, perlite, vermiculite, and coarse sand, at a ratio of 2:1:1:1 (v/v), and watered with half-strength Hoagland’s nutrient solution (Hoagland and Arnon, 1950). Plants were grown in a growth room under a 16/8 h light/dark regime at 24 °C. The light intensity was 100 μmol mol^–2^ s^–1^. The second fully expanded leaves of plants at the four true-leaf stage were used for measurements. The leaf mesophyll was isolated as described by Percey *et al.* (2016). Briefly, an appropriate leaf was excised, and the abaxial surface of the mesophyll was peeled off using very fine forceps. Leaf veins were prepared similarly and ion flux was measured at the cortex (see Supplementary Fig. S1 at *JXB* online). For the leaf mesophyll, peeled leaves were cut into 5 × 7 mm segments and for the leaf vein; peeled veins were cut into 3-cm long segments. These segments were then left floating (peeled side down) on BSM solution in 100 × 15-mm Petri dishes overnight prior to measurements to allow recovery from potentially confounding effects of wounding.

Net fluxes of K^+^ and Na^+^ were measured using the non-invasive microelectrode ion flux (MIFE) measurement technique as described previously (Cuin *et al.*, 2011; Wu *et al.*, 2015). For K^+^ flux measurements, the tips of the electrodes were front-filled with K^+^-selective cocktail (Sigma-Aldrich, catalog no. 99311). For Na^+^, recently developed calixarene-based microelectrodes with a superior Na^+^ selectivity were used (Jayakannan *et al.*, 2011).

For K^+^ flux measurements, ready-to-measure samples were immobilized in the measuring chamber and preconditioned in BSM for 30 min. During the measurements, a computer-controlled stepper motor moved the electrodes in a slow (6 s) square-wave cycle between the two positions, close to (40 μm for root, 130 μm from leaf mesophyll and vein) and further away (120 μm for root, 210 μm for leaf mesophyll and vein) from the measuring point. Steady-state ion fluxes were then recorded over a period of 5 min. Then, samples were treated with 100 mM NaCl or isotonic 170 mM sorbitol and the kinetics of net K^+^ fluxes were recorded for a further 30 min.

For Na^+^ measurements, a so-called ‘recovery protocol’ (Cuin *et al.*, 2011) was used to quantify the activity of the Na^+^ efflux system. The intact root, exposed leaf mesophyll, or isolated vein was treated with 100 mM NaCl for 24 h. The samples were then washed with 10 mM CaCl_2_ solution for 1 min to remove apoplastic Na^+^ and immobilized in the measuring chamber containing BSM solution. Measurements commenced 30 min after washing. By this time, all transient responses from the apoplast have ceased (see Cuin *et al.*, 2011, for supporting evidence), and the measured flux reflected the activity of the SOS1-mediated Na^+^ efflux system. Net Na^+^ flux was then measured over a 5–7 min interval.

### Agronomical and physiological assessments

For plants treated with 150 mM NaCl and harvested at day 15 of the salt treatment, dry weight was quantified after drying samples at 70 °C for 3 d. For elemental analysis, dried samples of appropriate tissues were digested in 10 ml 98% H_2_SO_4_ and 3 ml 30% H_2_O_2_, and then Na^+^ and K^+^ contents were measured using atomic absorption spectrophotometer (Varian spectra AA 220; Varian, Palo Alto, CA, USA).

For plants treated with 100 mM NaCl at the three true-leaf stage, gas exchange parameters were measured using an open gas-exchange system (Li-6400, Li-Cor, Inc., Lincoln, NE, USA) at 4, 24, and 120 h after NaCl or sorbitol treatment. The second fully expanded leaf was selected for measurements. Stomatal conductance (*g*_s_), transpiration rate (*T*_r_), and net photosynthetic rate (*P*_n_) were determined between 09.00 h and 12.00h. During measurements, the leaf chamber was controlled to maintain the leaf temperature at 25 °C, CO_2_ concentration at 360 μmol mol^–1^, and photosynthetic photon-flux density at 800 μmol m^–2^ s^–1^. The leaf RWC was calculated as [(fresh weight – dry weight)/(fresh weight at full turgor – dry weight)] ×100%.

### Quantification of Na^+^ in transverse sections of leaf veins by SEM-EDX

Samples were prepared as described by Lei *et al.* (2014), with a minor modification. The primary lateral vein of the second fully expanded leaf from the top of the plant was cut at day 10 after NaCl treatment (100 mM). Segments were dipped into 5% agar, inserted to a depth of 1.0 cm in a copper holder, immediately frozen in liquid nitrogen, and then hand-cut with a razor blade to obtain transverse sections. The samples were vacuum freeze-dried, carbon-coated in a high-vacuum sputter coater, and then analysed using a Hitachi S-3400N SEM-EDX (scanning electron microscopy and energy-dispersive X-ray; JSM-6390/LV; Horiba Ltd., Kyoto, Japan). The probe measurements of the segments were taken with a broad electron beam to analyse the relative elemental levels within the xylem parenchyma and cortex. Map scans were conducted by focusing a beam on the corresponding cells. Three transverse sections were observed for each treatment, and two location points on the same tissue of each section were analysed. The relative amount of Na^+^ was expressed as a percentage of the total atomic number for six major elements (K, Na, Cl, S, Ca, and Mg).

### Extraction and quantification of ABA

ABA content was measured in the second fully expanded leaf at 4, 24, and 120 h after NaCl treatment (100 mM at the three-leaf stage), following a previously described method (Wang *et al.*, 2012). Briefly, 0.5 g frozen root or leaf tissue was extracted in 4 ml of 80% methanol (v/v) containing 1 mM 2,6-di-*t*-butyl-*p*-cresol. The complete homogenate was incubated overnight in the dark at 4 °C. Following centrifugation (1000 *g* for 20 min), crude extract supernatants were filtered through a Sep-Pak C_18_ cartridge (Millipore, Milford, MA, USA) and dried under a N_2_ stream. Dried samples were suspended in 5 ml of elution buffer [10% (v/v) methanol in 50 mM Tris (pH 8.1), 1 mM MgCl_2_, 150 mM NaCl]. The samples were analysed using an immunoassay kit (China Agricultural University, Beijing, China) in accordance with the manufacturer’s instructions.

### Molecular analysis

For transcriptome analysis, representative genotypes of *C. maxima* and *C. moschata* (N12 and N15, respectively) were selected and grown hydroponically as described by Xie *et al.* (2015). The transcriptome responses of the leaf mesophyll and leaf veins were examined at 24 h after 100 mM NaCl treatment, using high-throughput RNA sequencing (RNA-seq). For quantitative real-time PCR (qRT-PCR) analysis, all 10 genotypes were used. The transcript levels of Na^+^ and K^+^ transporter genes (*HKT1*, *NHX4*, *NHX6*, *KUP6*, and *KEA6*), and the ABA synthesis gene *NCED3* were determined.

Total RNA was isolated using a TRIzol kit (Invitrogen, Carlsbad, CA, USA) with three biological replicates for each treatment. RNA concentration was measured using a Qubit® RNA Assay Kit in a Qubit® 2.0 Flurometer (Life Technologies, CA, USA). RNA integrity was assessed using the RNA Nano 6000 Assay Kit of the Bioanalyzer 2100 system (Agilent Technologies, CA, USA). Library construction and RNA-seq were conducted by Novogene Bioinformatics Institute (Beijing, China) on a HiSeq 4000 platform (Illumina, San Diego, CA, USA).

The clean data were obtained by removing low-quality reads from the raw data, and were mapped to the *Cucurbita maxima* (Rimu) and *Cucurbita moschata* (Rifu) genome assemblies (Sun *et al.*, 2017) using HISAT2 2.1.0 (Kim *et al.*, 2015). Fragments per kilobase of transcript sequence per million base pairs sequenced (FPKM) were calculated using featureCounts v1.6.0 (Liao *et al.*, 2014) to estimate gene expression levels. Differential expression analyses of RNA-seq between NaCl and control treatments, and between leaf vein and leaf mesophyll were performed using the DESeq2 package (Love *et al.*, 2014). The resulting *P*-values were adjusted using the Benjamini and Hochberg approach for controlling the false discovery rate. Genes with padj<0.05 and |log_2_ fold-change|>1 were assigned as differentially expressed genes (DEGs). The identified DEGs were then subjected to GO (gene ontology) and KEGG (Kyoto Encyclopedia of Genes and Genomes) pathway enrichment analyses using the GOseq R package and KOBAS 3.0 (Xie *et al.*, 2011), respectively. RNA-seq data were deposited in the National Center for Biotechnology Information (NCBI) Sequence Read Archive (SRA) under accession number PRJNA464060.

For qRT-PCR analysis, the PCR products were amplified using 1×Top Green qPCR SuperMix (TransGen Biotech, Inc., Beijing, China) in 10-μl qRT-PCR assays. The PCR was performed using an ABI 7000 (Applied Biosystems), and the cycling conditions consisted of denaturation at 94 °C for 30 s, followed by 40 cycles of denaturation at 95 °C for 5 s, annealing at 55 °C for 15 s, and extension at 72 °C for 15 s. The specific primers (listed in Supplementary Table S1) were designed based on published mRNA of *C. maxima* and *C. moschata* on the Cucurbit Genomics Database (http://cucurbitgenomics.org) using the Primer 5 software. The relative gene expression levels (transcript abundance) were expressed as relative quantification values calculated using the 2^−ΔΔ*C*t^ method (Livak and Schmittgen, 2001). Actin was used as the internal reference gene. For each genotype, the relative gene expression was determined as the gene expression under NaCl (100 mM NaCl) divided by the gene expression under control conditions (0 mM NaCl).

### Statistical analysis

Data were presented as means (±SE) of 3–6 biological replicates. Fisher’s LSD test was used to evaluate significant differences between treatments at *P*≤0.05. Statistical analyses were performed using SAS version 8.0 (SAS Institute Inc., Cary, NC, USA).

## Results

### *C. maxima* uses a mechanism of tissue tolerance to salinity

**Fig. 1.**
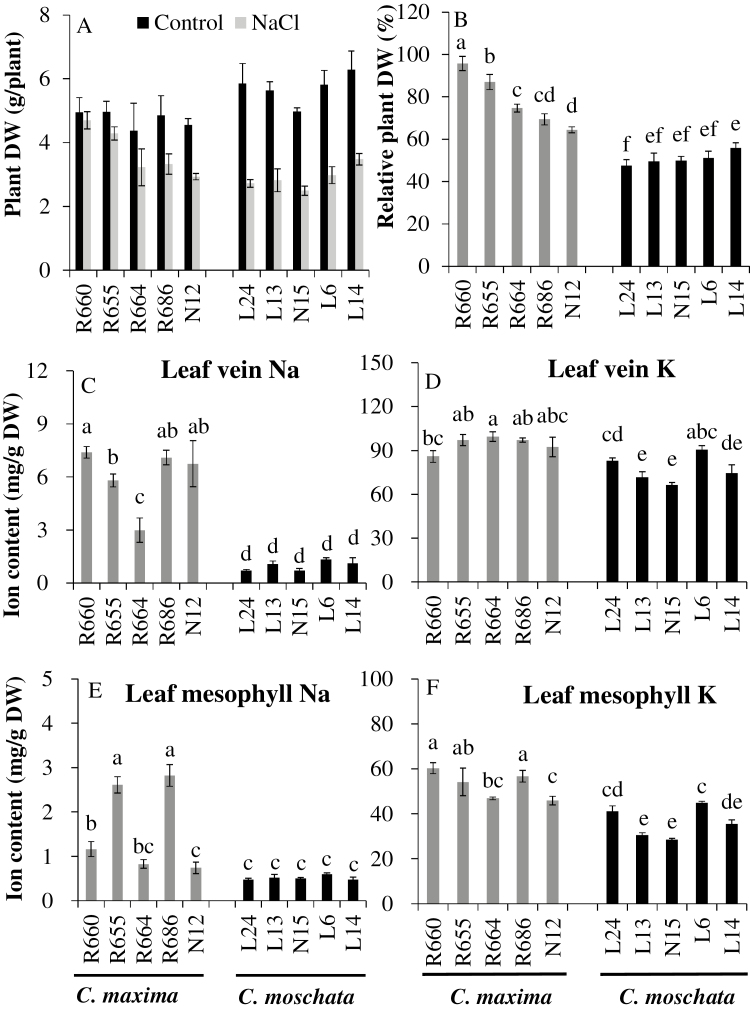
Plant dry weight (DW, A), relative plant DW (% of control) (B), Na^+^ and K^+^ contents in the leaf vein (C, D) and leaf mesophyll (E, F) for five *C. maxima* and five *C. moschata* genotypes grown in a substrate under 150 mM NaCl stress for 15 d. Values are means (±SE) (*n*=3). Different letters indicate significantly different values according to Fisher’s LSD test (*P*≤0.05).

Salinity stress decreased the plant dry weight of all genotypes in both species (Fig. 1A) but to differing extents (Fig. 1B). Overall, the relative dry weight (expressed as % of control) of salt-tolerant *C. maxima* was significantly higher than that of salt-sensitive *C. moschata* (78 ± 3% versus 51 ± 3%). At the same time, *C. maxima* accumulated more Na^+^ in the leaf vein, and also slightly more Na^+^ in the mesophyll compared with *C. moschata*, suggesting a typical tissue-tolerance mechanism (Fig. 1C, E). This was also supported by plant dry-weight measurements and leaf-vein Na^+^ accumulation after salt stress (Supplementary Fig. S2A–C).

Compared with the leaf mesophyll, both species had higher Na^+^ contents in the leaf veins, especially in *C. maxima* (Fig. 1C, E). The mean Na^+^ contents in the leaf veins and mesophyll of the five genotypes of *C. maxima* were 6.0 mg g^–1^ DW and 1.6 mg g^–1^ DW, respectively, while the corresponding values for *C. moschata* were 1.0 mg g^–1^ DW and 0.5 mg g^–1^ DW. The mean K^+^ contents in the leaf mesophyll of the five genotypes of *C. maxima* were higher than in *C. moschata* (53 mg g^–1^ DW versus 36 mg g^–1^ DW, respectively; Fig. 1F). This result was also supported by higher K^+^ accumulation in the leaf mesophyll under hydroponic growth conditions after salt stress (Supplementary Fig. S2D). The K^+^ content of leaf veins was also slightly higher in *C. maxima* (Fig. 1D).

### *C. maxima* can store more Na^+^ in the xylem parenchyma and cortex of the leaf vein than *C. moschata*

**Fig. 2.**
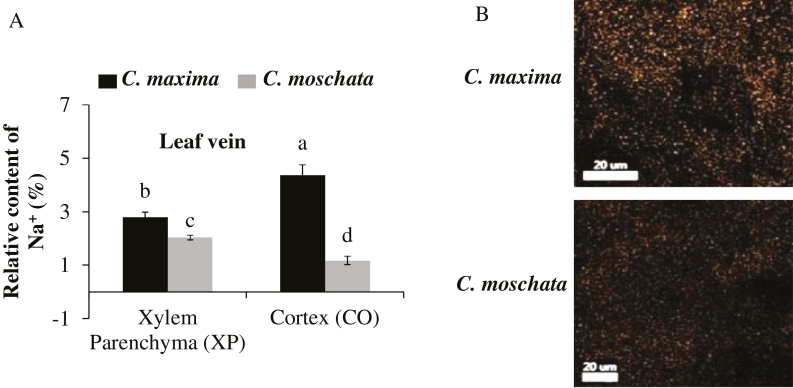
Relative Na^+^ content (A) measured by SEM-EDX (scanning electron microscopy and energy-dispersive X-ray microanalysis) in the xylem parenchyma and cortex in the leaf vein of plants of *C. maxima* (N12) and *C. moschata* (N15) grown hydroponically for 10 d under 100 mM NaCl treatment. Two representative images of the cortex Na^+^ distribution in the leaf vein from map-scanning are shown in (B). Values are means (±SE) (*n*=3). Different letters indicate significantly different values according to Fisher’s LSD test (*P*≤0.05).

The relative Na^+^ contents in the xylem parenchyma and cortex of the leaf veins were investigated using SEM-EDX. The results showed that *C. maxima* accumulated more Na^+^ in both than *C. moschata* under NaCl stress (Fig. 2A, B). The relative Na^+^ content in the cortex of the leaf veins of *C. maxima* was 3.7-fold higher compared with *C. moschata* (Fig. 2A).

### *C. maxima* has higher K^+^ retention in roots and leaf tissues that does not rely on Na^+^ exclusion from roots

**Fig. 3.**
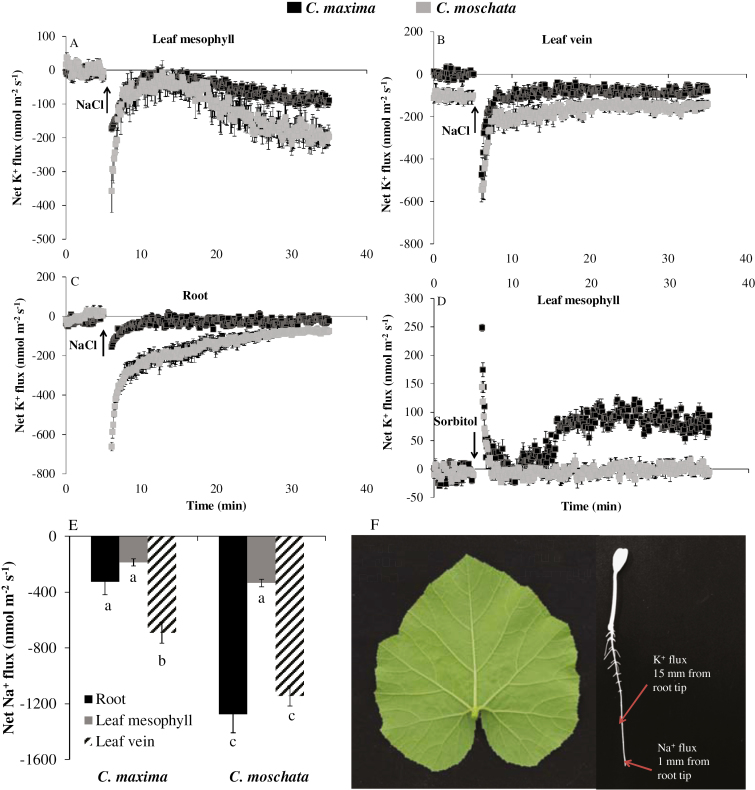
Transient net K^+^ flux measured from the leaf mesophyll (A), leaf veins (B), and roots (C) of plants of *C. maxima* (N12) and *C. moschata* (N15) in response to treatment with 100 mM NaCl. (D) Net K^+^ flux measured from the leaf mesophyll in response to treatment with isotonic 170 mM sorbitol. (E) Net Na^+^ flux from root epidermis, leaf mesophyll, and leaf veins of salt-stressed plants (100 mM NaCl for 24 h) measured 25 min after their transfer to Na-free solution. The measuring points for the Na^+^ and K^+^ fluxes are shown in (F). Values are means (±SE) (*n*=6). Different letters indicate significantly different values according to Fisher’s LSD test (*P*≤0.05). The sign convention for all the flux measurements is ‘efflux negative’.

The MIFE technique was used to quantify the differences in K^+^ retention and Na^+^ exclusion between *C. maxima* and *C. moschata* plants. The results showed that transient NaCl stress led to a massive K^+^ efflux from the leaf mesophyll (Fig. 3A), leaf vein (Fig. 3B), and root epidermis (Fig. 3C). However, the extent of K^+^ loss was significantly smaller in *C. maxima* than in *C. moschata*, indicating higher tissue tolerance in the latter species. This effect was specific for NaCl and was not observed in response to isotonic sorbitol treatment; when the leaf mesophyll was treated with isotonic sorbitol, net K^+^ influx was observed and was more pronounced in *C. maxima* (Fig. 3D).

To quantify the role of SOS1-mediated root Na^+^ exclusion, the so-called ‘recovery protocol’ was used (Cuin *et al* 2011). When plants were transferred to Na^+^-free BSM solution, net Na^+^ efflux was greater in *C. moschata* than in *C. maxima*, from both the root epidermis and the leaf vein tissue, while no significant difference was observed in the leaf mesophyll (Fig. 3E). The specific locations of the measuring points on the roots, leaf mesophyll and veins are shown in Fig. 3F. Overall, the results indicated that the better performance of *C. maxima* under saline conditions was not related to superior exclusion of Na^+^ from uptake.

### *C. maxima* maintains higher leaf water content due to rapid ABA accumulation inducing early stomatal closure under NaCl stress

**Fig. 4.**
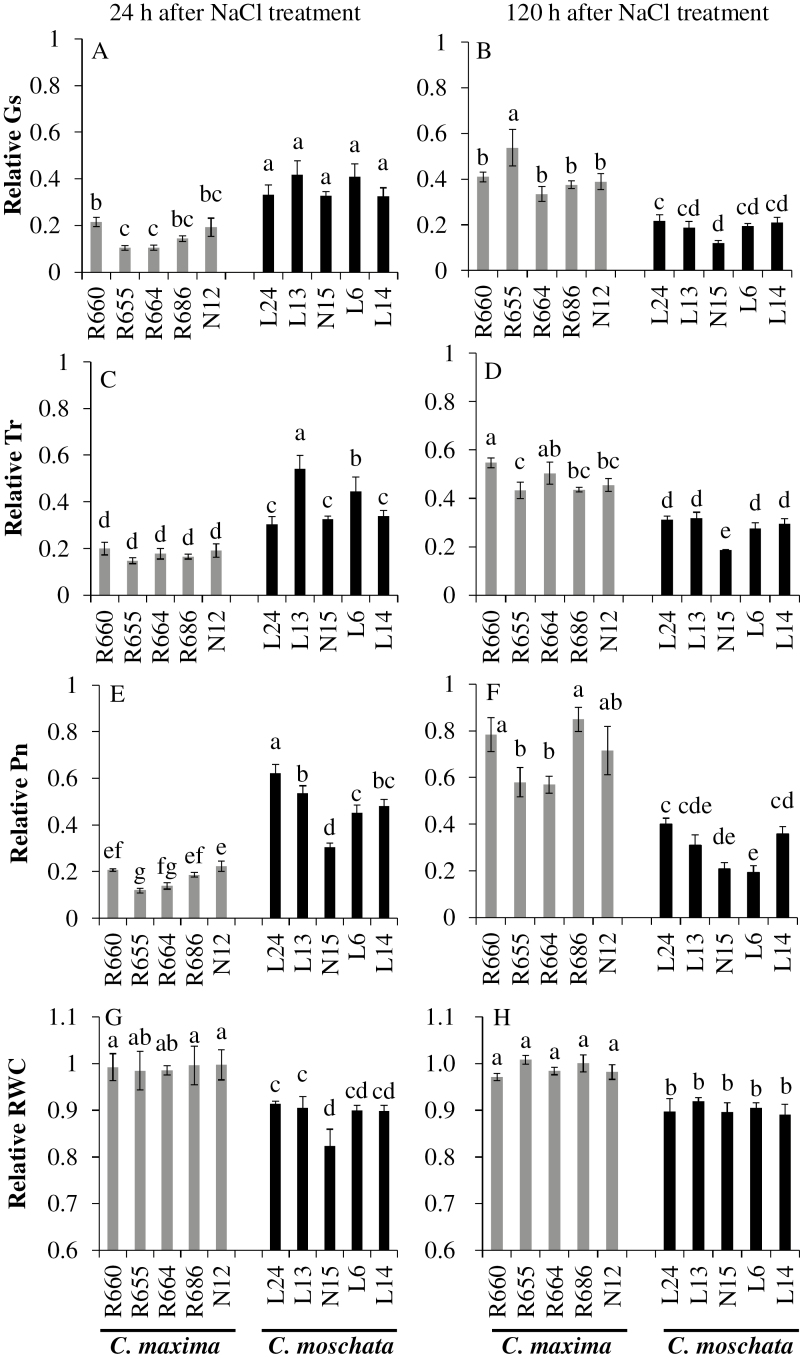
Relative values (NaCl/Control) for (A, B) leaf stomatal conductance (*g*_s_), (C, D) transpiration rate (*T*_r_), (E, F) net photosynthetic rate (*P*_n_), and (G, H) relative water content (RWC) for five *C. maxima* and five *C. moschata* genotypes grown hydroponically for either 24 h or 120 h under 100 mM NaCl treatment. Values are means (±SE) (*n*=4). Different letters indicate significantly different values according to Fisher’s LSD test (*P*≤0.05).

Stomatal conductance (*g*), transpiration rate (*T*_r_), and net photosynthetic rate (*P*_n_) (Supplementary Fig. S3) were significantly reduced in *C. maxima* 24 h after application of NaCl stress, while the relatives value in *C. moschata* were much higher (Fig. 4A, C, E). After 120 h of NaCl treatment, however, the relative values of *g*_s_, *T*_r_, and *P*_n_ were significantly higher in *C. maxima* than in *C. moschata* (Fig. 4B, D, F; Supplementary Fig. S3). These findings indicated that *C. maxima* closed its stomata at an early stage of the salt treatment and re-opened them at a later stage. Observations of stomatal apertures by SEM at 4 h after NaCl stress also supported this (Supplementary Fig. S4). As a result, *C. maxima* could maintain higher leaf RWC than *C. moschata* (Fig. 4G, H; Supplementary Fig. S).

**Fig. 5.**
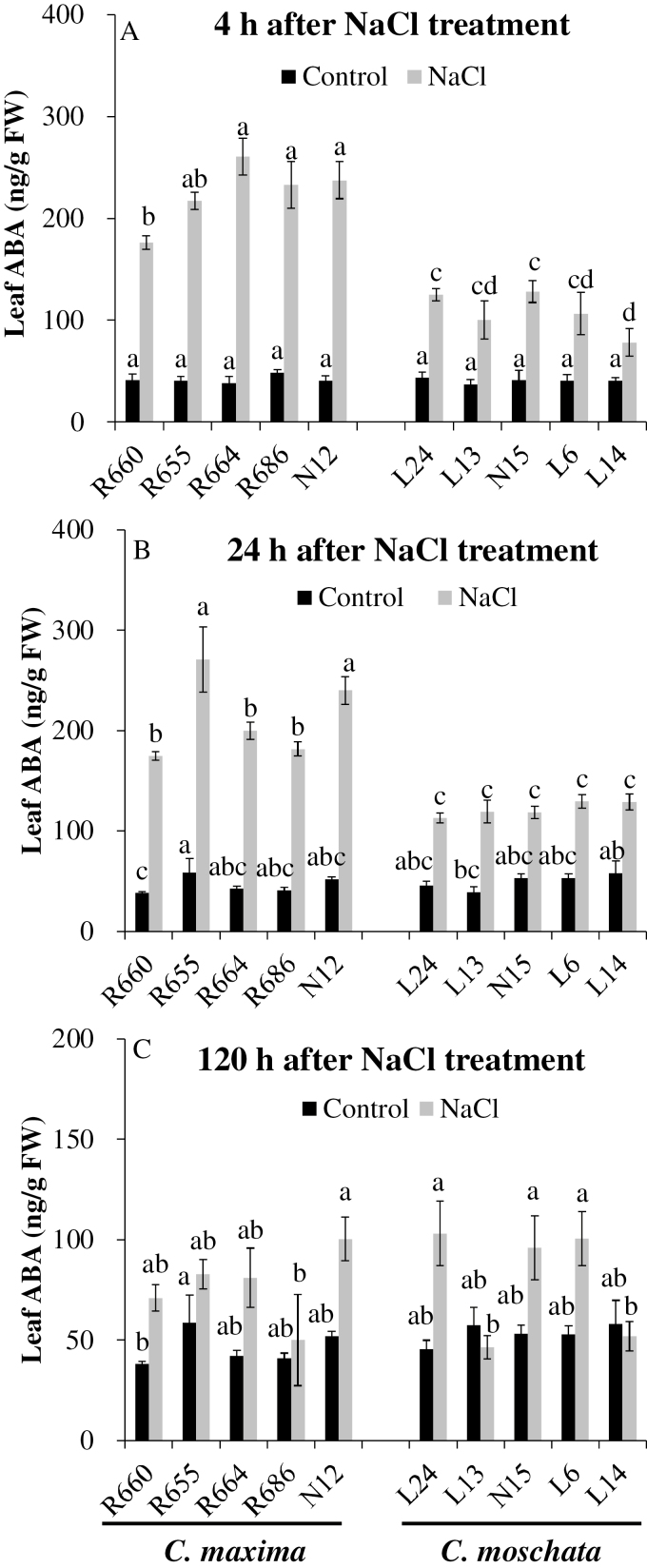
Leaf ABA concentrations for five *C. maxima* and five *C. moschata* genotypes grown hydroponically for 4 h (A), 24 h (B), or 120 h (C) under 100 mM NaCl treatment. Values are means (±SE) (*n*=4). Different letters indicate significantly different values between genotypes under control and NaCl stress (*P*≤0.05) according to Fisher’s LSD test.

To determine why the stomata closed so quickly, ABA concentrations were measured in the leaves (Fig. 5). The concentrations in *C. maxima* were significantly higher than those of *C. moschata* at 4 h and 24 h of NaCl stress (Fig. 5A, B). However, no consistent trend between the two species was observed at 120 h of treatment (Fig. 5C).

### Rapid stomatal closure in *C. maxima* is mainly due to the osmotic component of salinity stress

**Fig. 6.**
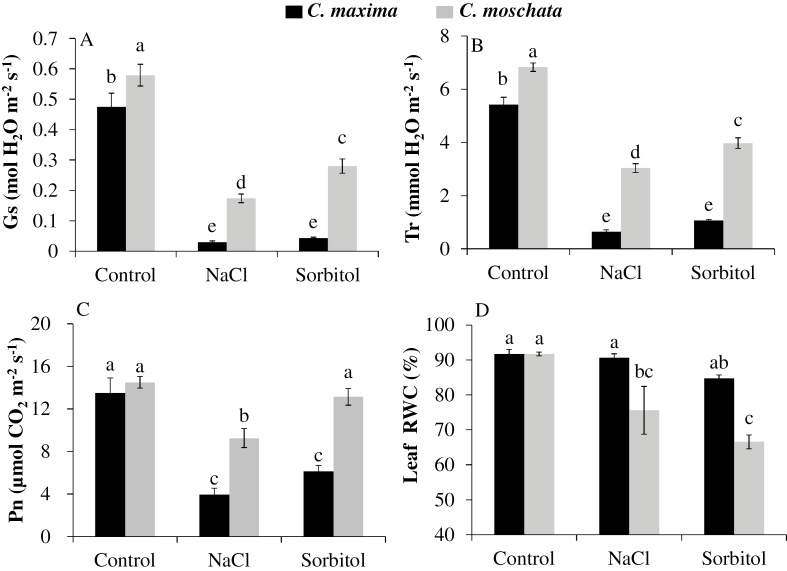
(A) Leaf stomatal conductance (*g*_s_), (B) transpiration rate (*T*_r_), (C) net photosynthetic rate (*P*_n_), and (D) relative water content (RWC) of *C. maxima* (N12) and *C. moschata* (N15) treated with NaCl (100 mM) or isotonic sorbitol (170 mM) solutions for 4 h. Values are means (±SE) (*n*=5). Different letters indicate significantly different values according to Fisher’s LSD test (*P*≤0.05).

To distinguish whether the rapid NaCl-induced stomatal closure in *C. maxima* was due to the ionic or osmotic component of salt stress, experiments using isotonic sorbitol treatments were conducted. The results showed that *C. maxima* had lower *g*_s_, *T*_r_, and *P*_n_ but higher leaf RWC compared with *C. moschata* after being exposed to either NaCl or isotonic sorbitol for 4 h (Fig. 6). These results suggested that the rapid stomatal closure observed in *C. maxima* could be mainly attributed to the osmotic component of the salt stress.

### Transcriptome and qRT-PCR analysis reveals the molecular mechanism involved in tissue salt tolerance in *C. maxima*

**Fig. 7.**
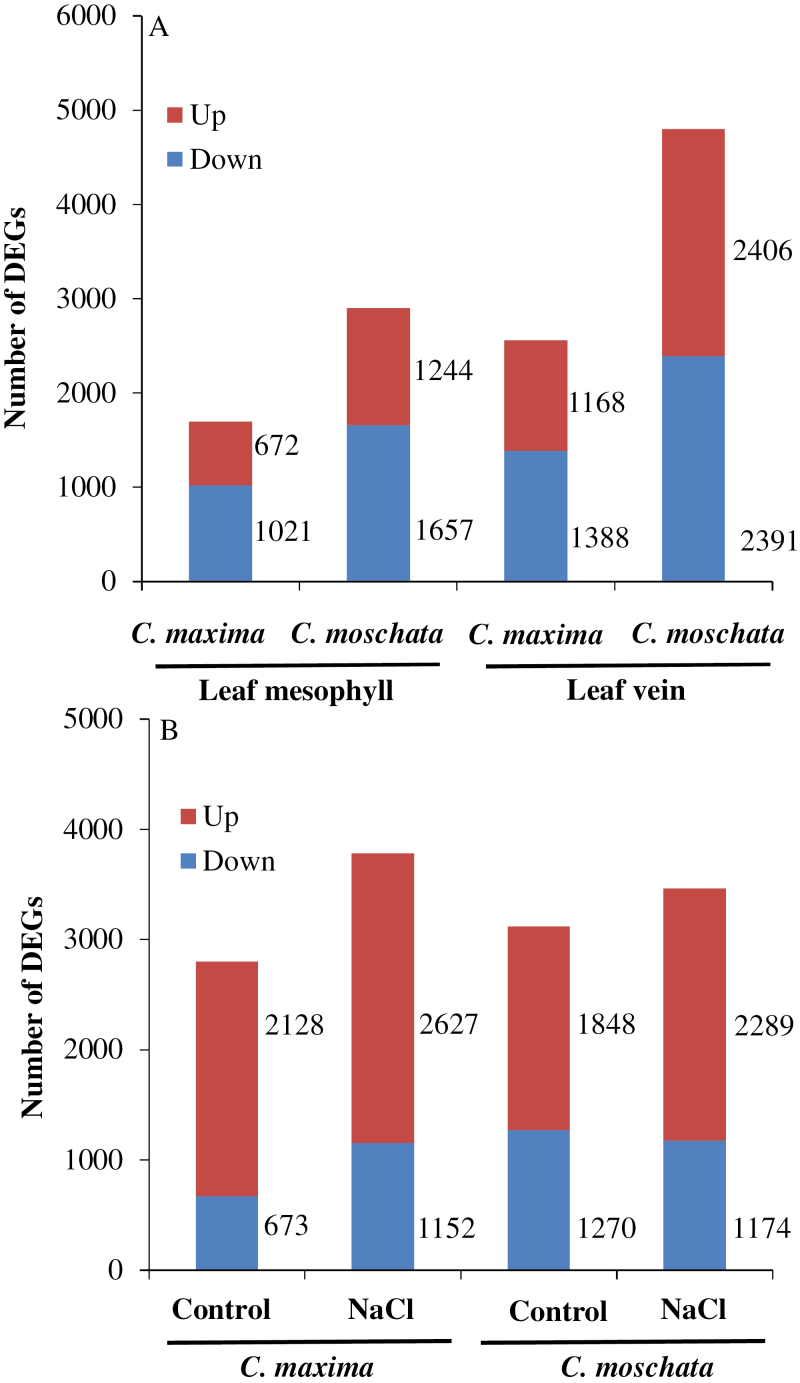
(A) Number of differentially expressed genes (DEGs) in the leaf mesophyll and leaf veins of *C. maxima* (N12) and *C. moschata* (N15) in response to 100 mM NaCl treatment for 24 h. (B) Number of DEGs of leaf veins compared with leaf mesophyll under control and NaCl conditions in *C. maxima* (N12) and *C. moschata* (N15).

To obtain further insights into the molecular mechanism of tissue tolerance to salinity in *Cucurbita*, the transcriptomic profiles between NaCl treatment and controls (Supplementary Tables S2–5) and between the leaf veins and leaf mesophyll (Supplementary Tables S6–9) were characterized. In *C. maxima* exposed to salinity, a total of 1693 (672 up, 1021 down) and 2556 (1168 up, 1388 down) DEGs were identified in the leaf mesophyll and leaf veins, respectively. The corresponding values for *C. moschata* were 2901 (1244 up, 1657 down) and 4797 (2406 up, 2391 down) (Fig. 7A) suggesting that *C. moschata* was more sensitive to salinity than *C. maxima* at the transcriptome level. Meanwhile, a total of 2801 (2128 up, 673 down) and 3779 (2627 up, 1152 down) DEGs were identified in the leaf veins of *C. maxima* under control conditions and NaCl stress, respectively, compared with leaf mesophyll. The corresponding values for *C. moschata* were 3118 (1848 up, 1270 down) and 3463 (2289 up, 1174 down) (Fig. 7B). These DEGs were further used for analyses of GO terms (Supplementary Tables S10–17) and the KEGG pathway (Supplementary Tables S18, 19).

**Fig. 8.**
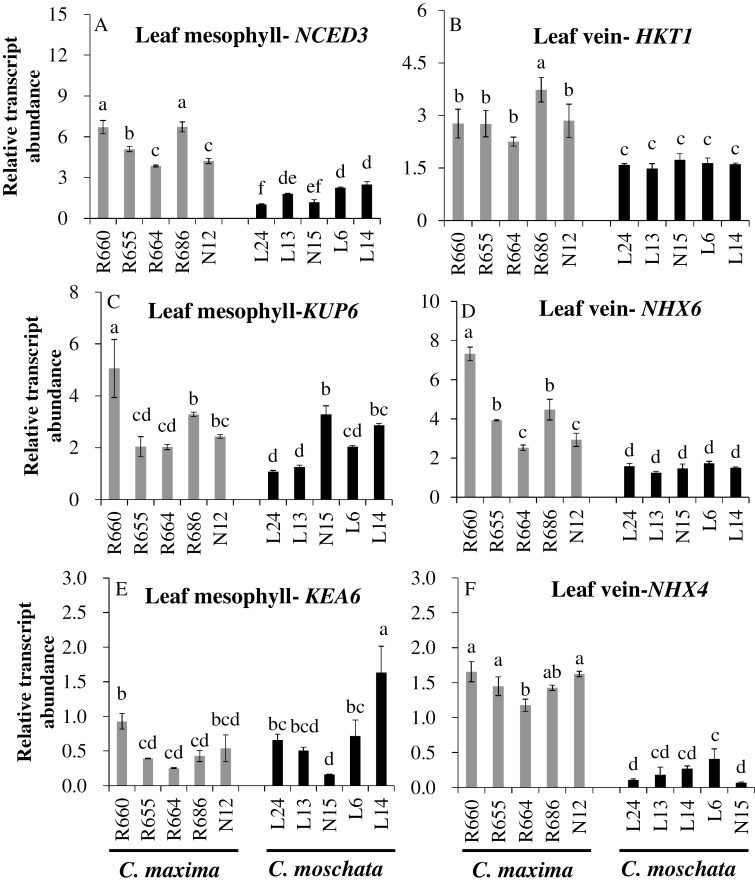
Relative expression of *NCED3* (A), *KUP6* (C), and *KEA6* (E) in the leaf mesophyll, and *HKT1* (B), *NHX6* (D), and *NHX4* (F) in the leaf veins of *C. maxima* and *C. moschata* genotypes grown hydroponically for 24 h under 100 mM NaCl stress. Values are means (±SE) (*n*=4). Different letters indicate significantly different values according to Fisher’s LSD test (*P*≤0.05).

A range of genes directly involved in Na^+^ and K^+^ transport and in ABA synthesis in the leaf mesophyll and leaf veins were significantly affected by salt stress in the two species examined in detail (Table 1). Salinity significantly increased the expression of *HKT1* (CmaCh10G003540) and *NHX6* (CmaCh13G003240) in the leaf veins of *C. maxima* but not in *C. moschata*. Meanwhile, salinity significantly decreased the expression of *NHX4* (CmoCh01G011470) in the leaf veins of *C. moschata* but not in *C. maxima*. Salinity also increased the expression of *NCED3*s (encoding 9-cis–epoxycarotenoid dioxygenase) in the leaf mesophyll in both species; however, more had increased expression in *C. maxima* (three DEGs, namely CmaCh16G004600, CmaCh04G006430, and CmaCh07G001000, compared with two DEGS in *C. moschata*, namely CmoCh04G006910 and CmoCh16G004950). qRT-PCR analysis was used to determine the expression of *NCED3*s in the leaf mesophyll (Fig. 8A), and this corresponded to the more rapid ABA signaling that was observed (Fig. 5A, B). In addition, expression of *HKT1* (Fig. 8B), *NHX6* (Fig. 8D), and *NHX4* (Fig. 8F) in the leaf veins was significantly higher in *C. maxima* than *C. moschata*, explaining the better tissue tolerance to salinity in the former. No consistent trend was observed for the expression of *KUP6* (Fig. 8C) and *KEA6* (Fig. 8E) in the leaf mesophyll, indicating that K^+^ retention rather than uptake may be a critical determinant of the differential K^+^ content observed in this tissue in both species (Fig. 1F; Supplementary Fig. S2D).

**Table 1.** Significant differentially expressed genes (DEGs; padj<0.05, |log_2_ fold-change|>1) involved in Na^+^ and K^+^ transport, and ABA synthesis in the transcriptome of *C. maxima* (N12) and *C. moschata* (N15) under NaCl stress (100 mM) compared with control conditions (0 mM NaCl)

ID	Log_2_ fold-change	Annotation	Arabidopsis homolog
Leaf mesophyll of *C. maxima*			
CmaCh01G011010	–1.78	Sodium/hydrogen exchanger	At5g55470/*NHX4*
CmaCh04G010720	–1.07	Potassium transporter	At2g40540/*KUP2*
CmaCh11G014180	1.10	Potassium transporter	At1g70300/*KUP6*
CmaCh12G006360	–1.19	Glutathione-regulated potassium-efflux system protein	At5g11800/*KEA6*
CmaCh16G004600	2.75	9-cis-epoxycarotenoid dioxygenase	At3g14440/*NCED3*
CmaCh04G006430	2.51	9-cis-epoxycarotenoid dioxygenase	At3g14440/*NCED3*
CmaCh07G001000	1.80	9-cis-epoxycarotenoid dioxygenase	At3g14440/*NCED3*
Leaf mesophyll of *C. moschata*			
CmoCh01G011470	–1.29	Sodium/hydrogen exchanger	At5g55470/*NHX4*
CmoCh01G007100	–1.66	Potassium transporter	At2g30070/*KUP1*
CmoCh11G017020	1.17	Potassium transporter	At1g70300/*KUP6*
CmoCh12G005760	–1.78	Glutathione-regulated potassium-efflux system protein	At5g11800/*KEA6*
CmoCh18G005760	2.21	Outward rectifying potassium channel protein	At5g55630/*TPK1*
CmoCh04G006910	4.21	9-cis-epoxycarotenoid dioxygenase	At3g14440/*NCED3*
CmoCh16G004950	1.26	9-cis-epoxycarotenoid dioxygenase	At3g14440/*NCED3*
Leaf vein of *C. maxima*			
CmaCh10G003540	1.02	Membrane Na^+^ transporter	At4g10310/*HKT1*
CmaCh13G003240	1.12	Sodium/hydrogen exchanger	At1g79610/*NHX6*
CmaCh11G014180	1.41	Potassium transporter	At1g70300/*KUP6*
CmaCh12G006360	–1.57	Glutathione-regulated potassium-efflux system protein	At5g11800/*KEA6*
CmaCh18G004630	1.79	Potassium channel	At5g46240/*KAT1*
CmaCh03G014040	1.36	9-cis-epoxycarotenoid dioxygenase	At3g14440/*NCED3*
Leaf vein of *C. moschata*			
CmoCh01G011470	–2.04	Sodium/hydrogen exchanger	At5g55470/*NHX4*
CmoCh01G007100	–2.19	Potassium transporter	At2g30070/*KUP1*
CmoCh06G011200	–1.19	Glutathione-regulated potassium-efflux system protein	At4g00630/*KEA2*
CmoCh11G014470	–1.25	Glutathione-regulated potassium-efflux system protein	At4g04850/*KEA3*
CmoCh12G005760	–1.81	Glutathione-regulated potassium-efflux system protein	At5g11800/*KEA6*
CmoCh18G005760	1.47	Outward rectifying potassium channel protein	At5g55630/*TPK1*
CmoCh04G005660	–1.25	Potassium voltage-gated channel subfamily H	At4g22200/*AKT2*
CmoCh16G004130	–1.21	Potassium voltage-gated channel subfamily H member	At4g22200/*AKT2*
CmoCh04G001080	–1.15	Potassium transporter	At3g56290/unchracterized protein
CmoCh03G013970	–1.96	9-cis-epoxycarotenoid dioxygenase	At3g14440/*NCED3*
CmoCh04G006910	4.34	9-cis-epoxycarotenoid dioxygenase	At3g14440/*NCED3*
CmoCh07G001020	2.46	9-cis-epoxycarotenoid dioxygenase	At3g14440/*NCED3*
CmoCh16G004950	1.81	9-cis-epoxycarotenoid dioxygenase	At3g14440/*NCED3*

## Discussion

### The tissue-tolerance mechanism is essential for salinity tolerance in *Cucurbita*

Many studies have suggested that excessive Na^+^ accumulation in the shoot leads to serious damage and eventual death (Munns and Tester, 2008). Our previous research found that some genotypes in *Cucurbita maxima* and *C. moschata* exhibited different Na^+^ accumulation patterns, with *C. maxima* accumulating more Na^+^ in the shoot (Xie *et al.*, 2015; Niu *et al.*, 2017). These findings have been further supported in this study following a comprehensive analysis of 10 *Cucurbita* genotypes. Despite having a much higher leaf Na^+^ content (Fig. 1C, E; Supplementary Fig. S2C), *C. maxima* clearly outperformed *C. moschata* when grown under saline conditions (Fig. 1A, B; Supplementary Fig. S2A, B). Plants of *C. maxima* also showed smaller increase in malonaldehyde (MDA) content (indicative of oxidative damage to membranes induced by NaCl), had lower H_2_O_2_ concentrations, and were able to maintain more optimal values for the maximum quantum yield of PSII (*F*_v_/*F*_m_) and the quantum efficiency of electron transfer at PSII (ΦPSII) (Supplementary Fig. S5). These findings suggest that the tissue-tolerance mechanism is essential for salinity tolerance in *Cucurbita*.

### Na^+^ accumulation in the leaf vein facilitates Na^+^ exclusion from the leaf mesophyll

As a major toxic ion, Na^+^ moves sequentially from the roots to the shoot, along the leaf petiole, and then spreads via the veins throughout the leaf. In this study, the Na^+^ content in the leaf vein was clearly higher than in the leaf mesophyll in both species (Fig. 1C, E), indicating that the strategy of restricting Na^+^ in leaf vein is commonly adopted by *Cucurbita*. The ability to restrict Na^+^ to the veins differed between *C. maxima* and *C. moschata*, with the former having a substantially higher content than the latter (Fig. 1C; Supplementary Fig. S2C), suggesting an important role of the leaf vein for storage of excessive salt loads. A similar phenomenon has been observed in leaves of banana, in which marginal veins display considerable resistance to Na^+^ flow from the xylem to the adjacent mesophyll (Shapira *et al.*, 2009). Other species such as rice have also been shown to adopt a similar strategy, depositing salt in the leaf sheath (Kobayashi *et al.*, 2017). This ability to restrict Na^+^ transport to the photosynthetic tissues is known to be an important adaptation to salinity stress (Munns and Tester, 2008).

### HKT1 in the leaf vein mediates Na^+^ exclusion from the mesophyll

Higher Na^+^ levels were found in the leaf veins than in the cortex and xylem parenchyma of the salt-tolerant *C. maxima* compared with the salt-sensitive *C. moschata* (Fig. 2A, B). Plant cortical cells have greater ion storage potential because they are more highly vacuolated than other cell types (Møller *et al.*, 2009). Moreover, xylem parenchyma has been reported to serve as a storage location for excess Na^+^ retrieved from the xylem vessels (Sunarpi *et al.*, 2005; Alemán *et al.*, 2011; Lin *et al.*, 2016). This Na^+^ retrieval process from the xylem vessels is mediated by HKT1, which is localized in xylem parenchyma cells and has been well studied in Arabidopsis, rice, and wheat (Horie *et al.*, 2009). A salinity-tolerant *japonica* rice cultivar was shown to have a Na^+^ exclusion mechanism in the leaf sheaths through the function of the Na^+^ transporter OsHKT1;4 under salinity stress (Wangsawang *et al.*, 2018). It has also been also reported that *ZmHKT1* promotes leaf Na^+^ exclusion and salt tolerance by withdrawing Na^+^ from the xylem sap in maize (Zhang *et al.*, 2018). In our study, higher Na^+^ accumulation in the xylem parenchyma of leaf veins in *C. maxima* was accompanied by higher expression of *HKT1* as determined in transcriptome and qRT-PCR experiments, suggesting a possible causal link between these two phenomena. In fact, both species had significantly higher *HKT1* expression in the leaf veins compared with the leaf mesophyll under NaCl stress (Supplementary Table S20), suggesting that leaf veins are essential for Na^+^ exclusion from the mesophyll.

### Vacuolar and endosomal compartments may play an important role in Na^+^ storage in the leaf veins of *C. maxima*

Intracellular Na^+^/H^+^ antiporters (NHXs) play important roles in many physiological processes, including cellular Na^+^ and K^+^ homeostasis in plants (Bassil *et al.*, 2011). NHXs are integral membrane proteins residing in the vacuoles (NHX1–4), endosomal compartments (NHX5, 6), and in the plasma membrane (NHX7/SOS1) (Bassil and Blumwald, 2014). Salinity significantly decreased the expression of *NHX4* in the leaf veins of *C. moschata* (Table 1, Fig. 8F), but not in *C. maxima* (Fig. 8F). Furthermore, the expression of *NHX4* was significantly higher in the leaf veins compared with the mesophyll under NaCl stress in *C. maxima* (Supplementary Table S20), suggesting that NHX4 may have been essential for the vacuolar sequestration of Na^+^. In addition, salinity significantly increased the expression of *NHX6* in the leaf veins of *C. maxima* but not in *C. moschata* (Table 1, Fig. 8D). Moreover, DEGs related to the membrane were significantly enriched between the leaf veins and the mesophyll under NaCl stress in GO terms, including intrinsic component of membrane (GO: 0031224), integral component of membrane (GO: 0016021), membrane part (GO: 0044425), transporter activity (GO: 0005215), and transmembrane transporter activity (GO: 0022857) (Supplementary Table S15). These results suggested that the vacuolar and endosomal compartments may play an important role in Na^+^ storage in the leaf veins of *C. maxima*.

Interestingly, higher Na^+^ efflux from roots and leaf veins in response to NaCl was observed in *C. moschata* than in *C. maxima* (Fig. 3E). In addition, using amiloride (a Na^+^/H^+^ antiporter inhibitor) as a pharmacological probe, we found that Na^+^ efflux from the roots of *C. moschata* and *C. maxima* decreased sharply (Supplementary Fig. S6), demonstrating that higher Na^+^ efflux in *C. moschata* could be mediated by higher activity of plasma-membrane Na^+^/H^+^ antiporters (Cuin *et al.*, 2011). However, SOS1, as an important component of Na^+^ efflux in the plasma membrane, may be regulated at the post-transcriptional and translational levels, as no significant differences were observed in the expression of *SOS1* under NaCl stress in either species (Table 1). Plants can save energy through a Na^+^ inclusion strategy, if the Na^+^ can be stored in an appropriate place such that it can reduce the amount of metabolic energy required for osmotic adjustment.

### The importance of leaf mesophyll K^+^ retention in the tissue tolerance of *Cucurbita* to salt

As one of the essential macronutrients, K plays important roles in many fundamental physiological processes in plant cells, including osmoregulation, enzyme activation, and ion homeostasis (Wang and Wu, 2015). It is also essential as a counter-ion for the charge balance of ion transport across the plasma- and intra-organelle membranes (Shabala, 2003). Therefore, a sufficient K supply is not only required for optimal plant growth and development, but also for plant stress tolerance. Under conditions of salt stress, membrane depolarization enables K^+^ efflux via outward-rectifying depolarization-activated K^+^ channels, shifting the overall K^+^ flux balance toward net efflux (Shabala and Cuin, 2008). Therefore, higher K^+^ retention is essential for plant function under saline conditions. An enhanced K^+^ retention and the ability of cells to maintain cytosolic K^+^ homeostasis correlate with salinity tolerance in a broad range of plant species (Yang *et al.*, 2013; Wu *et al.*, 2015; Chakraborty *et al.*, 2016*b*; Percey *et al.*, 2016; Shabala, 2017). Our study also demonstrated that the higher salt tolerance of *C. maxima* was related to higher K^+^ retention, especially in the leaf mesophyll (Figs 1F, 3A; Supplementary Fig. S2D). Interestingly, no consistent trends between the patterns of expression of the high-affinity K^+^ transporters *KUP6* and *KEA6* and the differential tolerance to salinity stress was found between the two species (Fig. 8C, E). This indicated that control of K^+^ leakage from the cell (mediated by either depolarization-activated GORK or ROS-activated NSCC channels; Shabala and Pottosin, 2014) but rather than K^+^ uptake may be a critical determinant of the differential K^+^ contents in the leaf mesophyll tissue in the two species.

The ionic and osmotic components of salt stress had different impacts on the plant K^+^ relations. NaCl stress induced a massive K^+^ efflux, while sorbitol treatment induced a K^+^ influx (Fig. 3D). A similar result was found previously in bean mesophyll tissue by Shabala (2000). The pattern of NaCl-induced fluxes of K^+^ differed from that caused by isotonic sorbitol in the leaf mesophyll, suggesting that they were mainly the result of ion-specific effects. In our study, lower H_2_O_2_ concentration and lipid peroxidation were observed in *C. maxima* (Supplementary Fig. S5C, D), probably due to the higher K^+^ retention and accumulation, since K^+^ is essential for preventing salinity-induced programmed cell death (Shabala, 2009; Demidchik *et al.*, 2010). In addition, salt-tolerant tissues have the ability to compartmentalize most of the Na^+^ within the vacuoles, rather than having any special tolerance of enzymes to high Na^+^ in the cytoplasm. The osmotic pressure in the cytoplasm would be balanced by K^+^ and organic solutes (Munns *et al.*, 2016).

### ABA accumulation regulated by NCED3 induces rapid stomatal closure and is an important component of tissue tolerance to salt in *Cucurbita*

In aerial plant tissues, maintaining proper water status requires the co-ordination of growth with the rate of water loss due to evapotranspiration and the water availability to the roots (Verslues *et al.*, 2006). Water stress in roots triggers a series of adjustments to plant physiology, including stomatal closure to limit evapotranspiration (Yamaguchi-Shinozaki and Shinozaki, 2006). These adjustments depend on the accumulation of ABA, a sesquiterpenoid plant hormone that has long been recognized as a key player in plant abiotic stress responses (Yamaguchi-Shinozaki and Shinozaki, 2006). Induced ABA accumulation early in osmotic stress can rapidly promote stomatal closure (MacRobbie, 1998), which can quickly reduce the transpiration rate and hence help plants to avoid water loss and maintain their water status. We also recently found that salinity tolerance of grafted cucumber was conferred by early stomatal closure (Niu *et al.*, 2018).

In this current study, rapid production of ABA was found in the leaves, resulting from the osmotic stress caused by salinity, which was consistent with a sharp decrease in stomatal conductance and transpiration at 4 h after NaCl treatment (Figs. 5A, 6A, B; Supplementary Fig. S4). The increase in ABA content and decrease in the gas-exchange parameters were greater in *C. maxima*, indicating that the quick stomatal closure was indeed regulated by the rapid ABA accumulation. As a result, the water status of *C. maxima* was improved compared with *C. moschata* (Fig. 4G, H).

Increases in ABA abundance in stressed tissues have been linked with the expression of one or more of the ABA biosynthetic genes, in particular the gene(s) encoding the 9-cis-epoxycarotenoid dioxygenase (NCED) enzyme (Speirs *et al.*, 2013). A major role of NCED3 in ABA production in response to stress has been reported in several studies (Hao *et al.*, 2009; Sussmilch *et al.*, 2017). In our study, higher ABA accumulation in the leaves of *C. maxima* could be attributed to the higher expression of *NCED3*s in the leaf mesophyll compared with the leaf veins under NaCl stress (Table 1; Supplementary Table S20). Recently, McAdam and Brodribb (2018) showed that the mesophyll cells are the main site of abscisic acid biosynthesis in water-stressed leaves.

Compared with *C. moschata*, *C. maxima* had a reduced photosynthetic rate during the early stage (24 h) of salt stress (Supplementary Fig. S3E); however, an obvious recovery in photosynthesis was observed in *C. maxima* from 120 h after salt stress (Supplementary Fig. S3F). In fact, the maximum quantum yield of PSII (*F*_v_/*F*_m_) and the quantum efficiency of electron transfer at PSII (ΦPSII) of *C. maxima* were significantly higher than those in *C. moschata* at day 10 after salt stress (Supplementary Fig. S5E–G). Thus, at later stages, *C. maxima* accumulated more biomass when compared with *C. moschata*.

## Conclusions

**Fig. 9.**
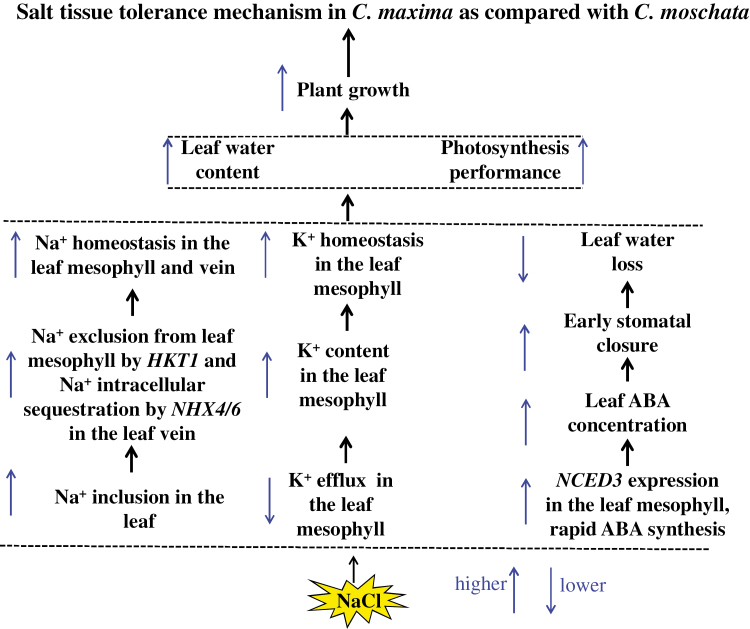
A simplified model depicting the tissue-tolerance mechanism to salinity of *C. maxima* as compared with *C. moschata*.

The results reported here demonstrate that *C. maxima* relies on the tissue-tolerance mechanism to combat salinity, while *C. moschata* species follow a salt-excluding strategy. Three complementary physiological traits confer the tissue-tolerance mechanism (Fig. 9). First, *C. maxima* species employ HKT1 to exclude Na^+^ from the leaf mesophyll and to keep it in the vein, where it is sequestered by NHX4/6. This allows the maintenance of Na^+^ homeostasis in the leaf mesophyll and veins, and also allows Na^+^ to be used as an energy-cheap osmoticum, thus reducing the cost of osmotic adjustment associated with accumulation of organic solutes. Second, NaCl stress induces a reduced K^+^ efflux from the roots and mesophyll of *C. maxima*. As a result, *C. maxima* plants retain more K^+^ in the leaf mesophyll and can maintain a normal level of cell metabolism. Third, NaCl stress induces a rapid accumulation of ABA in the leaves of *C. maxima* that is regulated by *NCED3*, leading to quick stomatal closure and thus avoiding excess water loss.

## Supplementary data

Supplementary data are available at *JXB* online.

Table S1. List of primer sequences used for qRT-PCR analysis

Table S2. Significant differentially expressed genes in the leaf mesophyll of *C. maxima* under NaCl stress compared with control conditions.

Table S3. Significant differentially expressed genes in the leaf vein of *C. maxima* under NaCl stress compared with control conditions.

Table S4. Significant differentially expressed genes in the leaf mesophyll of *C. moschata* under NaCl stress compared with control conditions.

Table S5. Significant differentially expressed genes in the leaf vein of *C. moschata* under NaCl stress compared with control conditions.

Table S6. Significant differentially expressed genes in the leaf vein compared with leaf mesophyll of *C. maxima* under control conditions.

Table S7. Significant differentially expressed genes in the leaf vein compared with leaf mesophyll of *C. maxima* under NaCl stress.

Table S8. Significant differentially expressed genes in the leaf vein compared with leaf mesophyll of *C. moschata* under control conditions.

Table S9. Significant differentially expressed genes in the leaf vein compared with leaf mesophyll of *C. moschata* under NaCl stress.

Table S10. Significant GO terms in the leaf mesophyll of *C. maxima* under NaCl stress compared with control conditions.

Table S11. Significant GO terms in the leaf vein of *C. maxima* under NaCl stress compared with control conditions.

Table S12. Significant GO terms in the leaf mesophyll of *C. moschata* under NaCl stress compared with control conditions.

Table S13. Significant GO terms in the leaf vein of *C. moschata* under NaCl stress compared with control conditions.

Table S14. Significant GO terms in the leaf vein compared with leaf mesophyll of *C. maxima* under control conditions.

Table S15. Significant GO terms in the leaf vein compared with leaf mesophyll of *C. maxima* under NaCl stress.

Table S16. Significant GO terms in the leaf vein compared with leaf mesophyll of *C. moschata* under control conditions.

Table S17. Significant GO terms in the leaf vein compared with leaf mesophyll of *C. moschata* under NaCl stress.

Table S18. Significant KEGG pathway enrichment under NaCl stress compared with control conditions in *C. maxima* and *C. moschata*.

Table S19. Significant KEGG pathway enrichment in the leaf vein compared with leaf mesophyll in *C. maxima* and *C. moschata*.

Table S20. Significant differentially expressed genes in the leaf vein transcriptome of *C. maxima* and *C. moschata* compared with the leaf mesophyll.

Fig. S1. Positions of measurement of ion fluxes in the leaf veins of *Cucurbita*.

Fig. S2. Plant dry weight (DW), relative DW, Na^+^ content in the leaf veins, and K^+^ content in the leaf mesophyll of *C. maxima* and *C. moschata* genotypes grown hydroponically under NaCl stress.

Fig. S3. Leaf stomatal conductance, transpiration rate, net photosynthetic rate, and relative water content of *C. maxima* and *C. moschata* genotypes grown hydroponically under NaCl stress.

Fig. S4. Images of leaf stomatal apertures for *C. maxima* (N12) and *C. moschata* (N15) grown hydroponically under NaCl stress.

Fig. S5. Growth, concentrations of leaf H_2_O_2_, and malonaldehyde, maximum quantum yield of PSII, and quantum efficiency of electron transfer of PSII for *C. maxima* (N12) and *C. moschata* (N15) grown hydroponically under control and NaCl stress conditions.

Fig. S6. Net Na^+^ flux from the root epidermis of *C. maxima* (N12) and *C. moschata* (N15) after removal of NaCl stress, with or without incubation with 100 μM amiloride.

## Supplementary Material

Supplementary Figures

Supplementary Tables S2-S5

Supplementary Tables S6-S19

## References

[CIT0001] Adolf VI, Jacobsen SE, Shabala S 2013 Salt tolerance mechanisms in quinoa (*Chenopodium quinoa* Willd.). Environmental and Experimental Botany 92, 43–54.

[CIT0002] Alemán F, Nieves-Cordones M, Martínez V, Rubio F 2011 Root K^+^ acquisition in plants: the *Arabidopsis thaliana* model. Plant & Cell Physiology 52, 1603–1612. 21771865 10.1093/pcp/pcr096

[CIT0003] Álvarez-Aragón R, Rodríguez-Navarro A 2017 Nitrate-dependent shoot sodium accumulation and osmotic functions of sodium in *Arabidopsis* under saline conditions. The Plant Journal 91, 208–219. 28370621 10.1111/tpj.13556

[CIT0004] Barragán V, Leidi EO, Andrés Z, Rubio L, De Luca A, Fernández JA, Cubero B, Pardo JM 2012 Ion exchangers NHX1 and NHX2 mediate active potassium uptake into vacuoles to regulate cell turgor and stomatal function in *Arabidopsis*. The Plant Cell 24, 1127–1142. 22438021 10.1105/tpc.111.095273 PMC3336136

[CIT0005] Bartha C, Fodorpataki L, del Carmen Martinez-Ballesta M, Popescu O, Carvajal M 2015 Sodium accumulation contributes to salt stress tolerance in lettuce cultivars. Journal of Applied Botany and Food Quality 88, 42–48.

[CIT0006] Bassil E, Ohto MA, Esumi T, Tajima H, Zhu Z, Cagnac O, Belmonte M, Peleg Z, Yamaguchi T, Blumwald E 2011 The *Arabidopsis* intracellular Na^+^/H^+^ antiporters NHX5 and NHX6 are endosome associated and necessary for plant growth and development. The Plant Cell 23, 224–239. 21278129 10.1105/tpc.110.079426 PMC3051250

[CIT0007] Bassil E, Blumwald E 2014 The ins and outs of intracellular ion homeostasis: NHX-type cation/H^+^ transporters. Current Opinion in Plant Biology 22, 1–6. 25173972 10.1016/j.pbi.2014.08.002

[CIT0008] Chakraborty K, Bose J, Shabala L, Eyles A, Shabala S 2016a Evaluating relative contribution of osmotolerance and tissue tolerance mechanisms toward salinity stress tolerance in three *Brassica* species. Physiologia Plantarum 158, 135–151. 27062083 10.1111/ppl.12447

[CIT0009] Chakraborty K, Bose J, Shabala L, Shabala S 2016b Difference in root K^+^ retention ability and reduced sensitivity of K^+^-permeable channels to reactive oxygen species confer differential salt tolerance in three *Brassica* species. Journal of Experimental Botany 67, 4611–4625. 27340231 10.1093/jxb/erw236 PMC4973732

[CIT0010] Colmenero-Flores JM, Martínez G, Gamba G, Vázquez N, Iglesias DJ, Brumós J, Talón M 2007 Identification and functional characterization of cation-chloride cotransporters in plants. The Plant Journal 50, 278–292. 17355435 10.1111/j.1365-313X.2007.03048.x

[CIT0011] Cuin TA, Bose J, Stefano G, Jha D, Tester M, Mancuso S, Shabala S 2011 Assessing the role of root plasma membrane and tonoplast Na^+^/H^+^ exchangers in salinity tolerance in wheat: *in planta* quantification methods. Plant, Cell & Environment 34, 947–961. 10.1111/j.1365-3040.2011.02296.x 21342209

[CIT0012] Demidchik V, Cuin TA, Svistunenko D, Smith SJ, Miller AJ, Shabala S, Sokolik A, Yurin V 2010 Arabidopsis root K^+^-efflux conductance activated by hydroxyl radicals: single-channel properties, genetic basis and involvement in stress-induced cell death. Journal of Cell Science 123, 1468–1479. 20375061 10.1242/jcs.064352

[CIT0013] Edelstein M, Plaut Z, Ben-Hur M 2011 Sodium and chloride exclusion and retention by non-grafted and grafted melon and *Cucurbita* plants. Journal of Experimental Botany 62, 177–184. 20729482 10.1093/jxb/erq255 PMC2993908

[CIT0014] Gálvez FJ, Baghour M, Hao G, Cagnac O, Rodríguez-Rosales MP, Venema K 2012 Expression of LeNHX isoforms in response to salt stress in salt sensitive and salt tolerant tomato species. Plant Physiology and Biochemistry 51, 109–115. 22153246 10.1016/j.plaphy.2011.10.012

[CIT0015] Genc Y, McDonald GK, Tester M 2007 Reassessment of tissue Na+ concentration as a criterion for salinity tolerance in bread wheat. Plant, Cell & Environment 30, 1486–1498. 10.1111/j.1365-3040.2007.01726.x 17897418

[CIT0016] Hao GP, Zhang XH, Wang YQ, Wu ZY, Huang CL 2009 Nucleotide variation in the *NCED3* region of *Arabidopsis thaliana* and its association study with abscisic acid content under drought stress. Journal of Integrative Plant Biology 51, 175–183. 19200156 10.1111/j.1744-7909.2008.00786.x

[CIT0017] Hoagland DR, Arnon DI 1950 The water culture method for growing plants without soil. California Agricultural Experiment Station Circular 347, 1–32.

[CIT0018] Horie T, Hauser F, Schroeder JI 2009 HKT transporter-mediated salinity resistance mechanisms in *Arabidopsis* and monocot crop plants. Trends in Plant Science 14, 660–668. 19783197 10.1016/j.tplants.2009.08.009 PMC2787891

[CIT0019] Huang Y, Bie ZL, Liu PY, et al 2013 Reciprocal grafting between cucumber and pumpkin demonstrates the roles of the rootstock in the determination of cucumber salt tolerance and sodium accumulation. Scientia Horticulturae 149, 47–54.

[CIT0020] Ismail A, Takeda S, Nick P 2014 Life and death under salt stress: same players, different timing?Journal of Experimental Botany 65, 2963–2979. 24755280 10.1093/jxb/eru159

[CIT0021] James RA, Munns R, von Caemmerer S, Trejo C, Miller C, Condon TA 2006 Photosynthetic capacity is related to the cellular and subcellular partitioning of Na^+^, K^+^ and Cl^-^ in salt-affected barley and durum wheat. Plant, Cell & Environment 29, 2185–2197. 10.1111/j.1365-3040.2006.01592.x 17081251

[CIT0022] Jayakannan M, Babourina O, Rengel Z 2011 Improved measurements of Na+ fluxes in plants using calixarene-based microelectrodes. Journal of Plant Physiology 168, 1045–1051. 21256620 10.1016/j.jplph.2010.12.006

[CIT0023] Kobayashi NI, Yamaji N, Yamamoto H, et al 2017 OsHKT1;5 mediates Na^+^ exclusion in the vasculature to protect leaf blades and reproductive tissues from salt toxicity in rice. The Plant Journal 91, 657–670. 28488420 10.1111/tpj.13595

[CIT0024] Kim D, Langmead B, Salzberg SL 2015 HISAT: a fast spliced aligner with low memory requirements. Nature Methods 12, 357–360. 25751142 10.1038/nmeth.3317 PMC4655817

[CIT0025] Kong QS, Chen JL, Liu Y, Ma YH, Liu P, Wu SY, Huang Y, Bie ZL 2014 Genetic diversity of *Cucurbita* rootstock germplasm as assessed using simple sequence repeat markers. Scientia Horticulturae 175, 150–155.

[CIT0026] Lei B, Huang Y, Sun J, Xie J, Niu M, Liu Z, Fan M, Bie Z 2014 Scanning ion-selective electrode technique and X-ray microanalysis provide direct evidence of contrasting Na^+^ transport ability from root to shoot in salt-sensitive cucumber and salt-tolerant pumpkin under NaCl stress. Physiologia Plantarum 152, 738–748. 24813633 10.1111/ppl.12223

[CIT0027] Liao Y, Smyth GK, Shi W 2014 featureCounts: an efficient general purpose program for assigning sequence reads to genomic features. Bioinformatics 30, 923–930. 24227677 10.1093/bioinformatics/btt656

[CIT0028] Lin KC, Jwo WS, Chandrika NNP, Wu TM, Lai MH, Wang CS, Hong CY 2016 A rice mutant defective in antioxidant-defense system and sodium homeostasis possesses increased sensitivity to salt stress. Biologia Plantarum 60, 86–94.

[CIT0029] Livak KJ, Schmittgen TD 2001 Analysis of relative gene expression data using real-time quantitative PCR and the 2^–△△*C*t^ method. Methods 25, 402–408. 11846609 10.1006/meth.2001.1262

[CIT0030] Love MI, Huber W, Anders S 2014 Moderated estimation of fold change and dispersion for RNA-seq data with DESeq2. Genome Biology 15, 550. 25516281 10.1186/s13059-014-0550-8 PMC4302049

[CIT0031] MacRobbie EA 1998 Signal transduction and ion channels in guard cells. Philosophical Transactions of the Royal Society of London. Series B, Biological Sciences 353, 1475–1488. 9800209 10.1098/rstb.1998.0303 PMC1692354

[CIT0032] McAdam SAM, Brodribb TJ 2018 Mesophyll cells are the main site of abscisic acid biosynthesis in water-stressed leaves. Plant Physiology. In press, doi:10.1104/pp.17.01829. PMC6052997 29735726

[CIT0033] Møller IS, Gilliham M, Jha D, Mayo GM, Roy SJ, Coates JC, Haseloff J, Tester M 2009 Shoot Na^+^ exclusion and increased salinity tolerance engineered by cell type-specific alteration of Na^+^ transport in *Arabidopsis*. The Plant Cell 21, 2163–2178. 19584143 10.1105/tpc.108.064568 PMC2729596

[CIT0034] Munns R, Gilliham M 2015 Salinity tolerance of crops – what is the cost?New Phytologist 208, 668–673. 26108441 10.1111/nph.13519

[CIT0035] Munns R, James RA, Gilliham M, Flowers TJ, Colmer TD 2016 Tissue tolerance: an essential but elusive trait for salt-tolerant crops. Functional Plant Biology 43, 1103–1113. 10.1071/FP16187 32480530

[CIT0036] Munns R, Tester M 2008 Mechanisms of salinity tolerance. Annual Review of Plant Biology 59, 651–681. 10.1146/annurev.arplant.59.032607.092911 18444910

[CIT0037] Niu ML, Huang Y, Sun ST, Sun JY, Cao HS, Shabala S, Bie ZL 2018 Root respiratory burst oxidase homologue-dependent H_2_O_2_ production confers salt tolerance on a grafted cucumber by controlling Na^+^ exclusion and stomatal closure. Journal of Experimental Botany 69, 3465–3476. 29145593 10.1093/jxb/erx386 PMC6009698

[CIT0038] Niu ML, Xie JJ, Sun JY, Huang Y, Kong QS, Nawaz MA, Bie ZL 2017 A shoot based Na^+^ tolerance mechanism observed in pumpkin—An important consideration for screening salt tolerant rootstocks. Scientia Horticulturae 218, 38–47.

[CIT0039] Olías R, Eljakaoui Z, Li J, De Morales PA, Marín-Manzano MC, Pardo JM, Belver A 2009 The plasma membrane Na^+^/H^+^ antiporter SOS1 is essential for salt tolerance in tomato and affects the partitioning of Na^+^ between plant organs. Plant, Cell & Environment 32, 904–916. 10.1111/j.1365-3040.2009.01971.x 19302170

[CIT0040] Percey WJ, Shabala L, Wu Q, Su N, Breadmore MC, Guijt RM, Bose J, Shabala S 2016 Potassium retention in leaf mesophyll as an element of salinity tissue tolerance in halophytes. Plant Physiology and Biochemistry 109, 346–354. 27810674 10.1016/j.plaphy.2016.10.011

[CIT0041] Prusty MR, Kim SR, Vinarao R, Entila F, Egdane J, Diaz MGQ, Jena KK 2018 Newly identified wild rice accessions conferring high salt tolerance might use a tissue tolerance mechanism in leaf. Frontiers in Plant Science 9, 417. 29740456 10.3389/fpls.2018.00417 PMC5926390

[CIT0042] Qadir M, Quillérou E, Nangia V, Murtaza G, Singh M, Thomas RJ, Drechsel P, Noble AD 2014 Economics of salt-induced land degradation and restoration. Natural Resources Forum 38, 282–295.

[CIT0043] Rahnama A, James RA, Poustini K, Munns R 2010 Stomatal conductance as a screen for osmotic stress tolerance in durum wheat growing in saline soil. Functional Plant Biology 37, 255–263.

[CIT0044] Rajendran K, Tester M, Roy SJ 2009 Quantifying the three main components of salinity tolerance in cereals. Plant, Cell & Environment 32, 237–249. 10.1111/j.1365-3040.2008.01916.x 19054352

[CIT0045] Santa-María GE, Oliferuk S, Moriconi JI 2018 KT-HAK-KUP transporters in major terrestrial photosynthetic organisms: a twenty years tale. Journal of Plant Physiology 226, 77–90. 29704646 10.1016/j.jplph.2018.04.008

[CIT0046] Shabala L, Zhang J, Pottosin I, et al 2016 Cell-type-specific H^+^-ATPase activity in root tissues enables K^+^ retention and mediates acclimation of barley (*Hordeum vulgare*) to salinity stress. Plant Physiology 172, 2445–2458. 27770060 10.1104/pp.16.01347 PMC5129721

[CIT0047] Shabala S 2000 Ionic and osmotic components of salt stress specifically modulate net ion fluxes from bean leaf mesophyll. Plant, Cell and Environment 23, 825–837.

[CIT0048] Shabala S 2003 Regulation of potassium transport in leaves: from molecular to tissue level. Annals of Botany 92, 627–634. 14500326 10.1093/aob/mcg191 PMC4244855

[CIT0049] Shabala S 2009 Salinity and programmed cell death: unravelling mechanisms for ion specific signalling. Journal of Experimental Botany 60, 709–712. 19269993 10.1093/jxb/erp013

[CIT0050] Shabala S 2017 Signalling by potassium: another second messenger to add to the list?Journal of Experimental Botany 68, 4003–4007. 28922770 10.1093/jxb/erx238 PMC5853517

[CIT0051] Shabala S, Bose J, Hedrich R 2014 Salt bladders: do they matter?Trends in Plant Science 19, 687–691. 25361704 10.1016/j.tplants.2014.09.001

[CIT0052] Shabala S, Cuin TA 2008 Potassium transport and plant salt tolerance. Physiologia Plantarum 133, 651–669. 18724408 10.1111/j.1399-3054.2007.01008.x

[CIT0053] Shabala S, Pottosin I 2014 Regulation of potassium transport in plants under hostile conditions: implications for abiotic and biotic stress tolerance. Physiologia Plantarum 151, 257–279. 24506225 10.1111/ppl.12165

[CIT0054] Shabala SN, Lew RR 2002 Turgor regulation in osmotically stressed Arabidopsis epidermal root cells. Direct support for the role of inorganic ion uptake as revealed by concurrent flux and cell turgor measurements. Plant Physiology 129, 290–299. 12011359 10.1104/pp.020005 PMC155892

[CIT0055] Shapira O, Khadka S, Israeli Y, Shani U, Schwartz A 2009 Functional anatomy controls ion distribution in banana leaves: significance of Na^+^ seclusion at the leaf margins. Plant, Cell & Environment 32, 476–485. 10.1111/j.1365-3040.2009.01941.x 19183293

[CIT0056] Shi H, Quintero FJ, Pardo JM, Zhu JK 2002 The putative plasma membrane Na^+^/H^+^ antiporter SOS1 controls long-distance Na^+^ transport in plants. The Plant Cell 14, 465–477. 11884687 10.1105/tpc.010371 PMC152925

[CIT0057] Speirs J, Binney A, Collins M, Edwards E, Loveys B 2013 Expression of ABA synthesis and metabolism genes under different irrigation strategies and atmospheric VPDs is associated with stomatal conductance in grapevine (*Vitis vinifera* L. cv Cabernet Sauvignon). Journal of Experimental Botany 64, 1907–1916. 23630325 10.1093/jxb/ert052 PMC3638820

[CIT0058] Sun H, Wu S, Zhang G, et al 2017 Karyotype stability and unbiased fractionation in the paleo-allotetraploid *Cucurbita* genomes. Molecular Plant 10, 1293–1306. 28917590 10.1016/j.molp.2017.09.003

[CIT0059] Sunarpi, Horie T, Motoda J, et al 2005 Enhanced salt tolerance mediated by AtHKT1 transporter-induced Na unloading from xylem vessels to xylem parenchyma cells. The Plant Journal 44, 928–938. 16359386 10.1111/j.1365-313X.2005.02595.x

[CIT0060] Sussmilch FC, Brodribb TJ, McAdam SAM 2017 Up-regulation of *NCED3* and ABA biosynthesis occur within minutes of a decrease in leaf turgor but *AHK1* is not required. Journal of Experimental Botany 68, 2913–2918. 28449122 10.1093/jxb/erx124 PMC5853609

[CIT0061] Verslues PE, Agarwal M, Katiyar-Agarwal S, Zhu J, Zhu JK 2006 Methods and concepts in quantifying resistance to drought, salt and freezing, abiotic stresses that affect plant water status. The Plant Journal 45, 523–539. 16441347 10.1111/j.1365-313X.2005.02593.x

[CIT0062] Wang SM, Zhang JL, Flowers TJ 2007 Low-affinity Na^+^ uptake in the halophyte *Suaeda maritima*. Plant Physiology 145, 559–571. 17766398 10.1104/pp.107.104315 PMC2048717

[CIT0063] Wang Y, Li B, Du M, Eneji AE, Wang B, Duan L, Li Z, Tian X 2012 Mechanism of phytohormone involvement in feedback regulation of cotton leaf senescence induced by potassium deficiency. Journal of Experimental Botany 63, 5887–5901. 22962680 10.1093/jxb/ers238 PMC3467299

[CIT0064] Wang Y, Wu WH 2015 Genetic approaches for improvement of the crop potassium acquisition and utilization efficiency. Current Opinion in Plant Biology 25, 46–52. 25941764 10.1016/j.pbi.2015.04.007

[CIT0065] Wangsawang T, Chuamnakthong S, Kohnishi E, Sripichitt P, Sreewongchai T, Ueda A 2018 A salinity‐tolerant japonica cultivar has Na^+^ exclusion mechanism at leaf sheaths through the function of a Na^+^ transporter OsHKT1; 4 under salinity stress. Journal of Agronomy and Crop Science 204, 274–284.

[CIT0066] Wu H, Zhu M, Shabala L, Zhou M, Shabala S 2015 K^+^ retention in leaf mesophyll, an overlooked component of salinity tolerance mechanism: a case study for barley. Journal of Integrative Plant Biology 57, 171–185. 25040138 10.1111/jipb.12238

[CIT0067] Xie C, Mao X, Huang J, et al 2011 KOBAS 2.0: a web server for annotation and identification of enriched pathways and diseases. Nucleic Acids Research 39, W316–W322. 21715386 10.1093/nar/gkr483 PMC3125809

[CIT0068] Xie J, Lei B, Niu M, Huang Y, Kong Q, Bie Z 2015 High throughput sequencing of small RNAs in the two *Cucurbita* germplasm with different sodium accumulation patterns identifies novel microRNAs involved in salt stress response. PloS ONE 10, e0127412. 26010449 10.1371/journal.pone.0127412 PMC4444200

[CIT0069] Yamaguchi-Shinozaki K, Shinozaki K 2006 Transcriptional regulatory networks in cellular responses and tolerance to dehydration and cold stresses. Annual Review of Plant Biology 57, 781–803. 10.1146/annurev.arplant.57.032905.105444 16669782

[CIT0070] Yang L, Zu YG, Tang ZH 2013 Ethylene improves Arabidopsis salt tolerance mainly via retaining K^+^ in shoots and roots rather than decreasing tissue Na^+^ content. Environmental and Experimental Botany 86, 60–69.

[CIT0071] Zaki HEM, Yokoi S 2016 A comparative *in vitro* study of salt tolerance in cultivated tomato and related wild species. Plant Biotechnology 33, 361–372. 10.5511/plantbiotechnology.16.1006a PMC6587036 31274997

[CIT0072] Zhang M, Cao Y, Wang Z, et al 2018 A retrotransposon in an HKT1 family sodium transporter causes variation of leaf Na^+^ exclusion and salt tolerance in maize. New Phytologist 217, 1161–1176. 29139111 10.1111/nph.14882

[CIT0073] Zheng S, Pan T, Fan L, Qiu QS 2013 A novel AtKEA gene family, homolog of bacterial K^+^/H^+^ antiporters, plays potential roles in K^+^ homeostasis and osmotic adjustment in *Arabidopsis*. PLoS ONE 8, e81463. 24278440 10.1371/journal.pone.0081463 PMC3835744

