# Supplementary material for: An early ABA-induced stomatal closure, Na+ sequestration in leaf vein and K+ retention in mesophyll confer salt tissue tolerance in Cucurbita species
Source: J Exp Bot. 2018 Jul 10;69(20):4945–60. doi: 10.1093/jxb/ery251 (PMC6137988; doi:10.1093/jxb/ery251)
Supplement: Supplementary Figures [file ery251_suppl_supplementary_figures.pdf]

Supplementary Figures

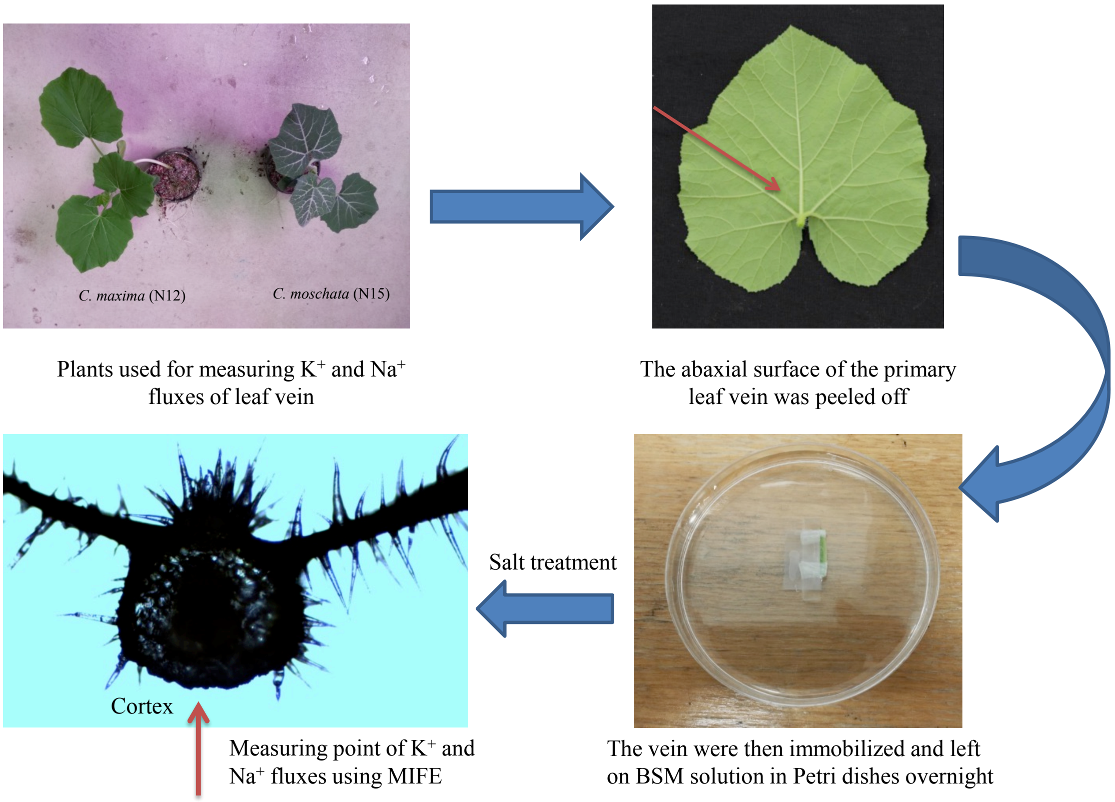

Fig. S1. Measured position of ion flux in the leaf vein of *Cucurbita*.

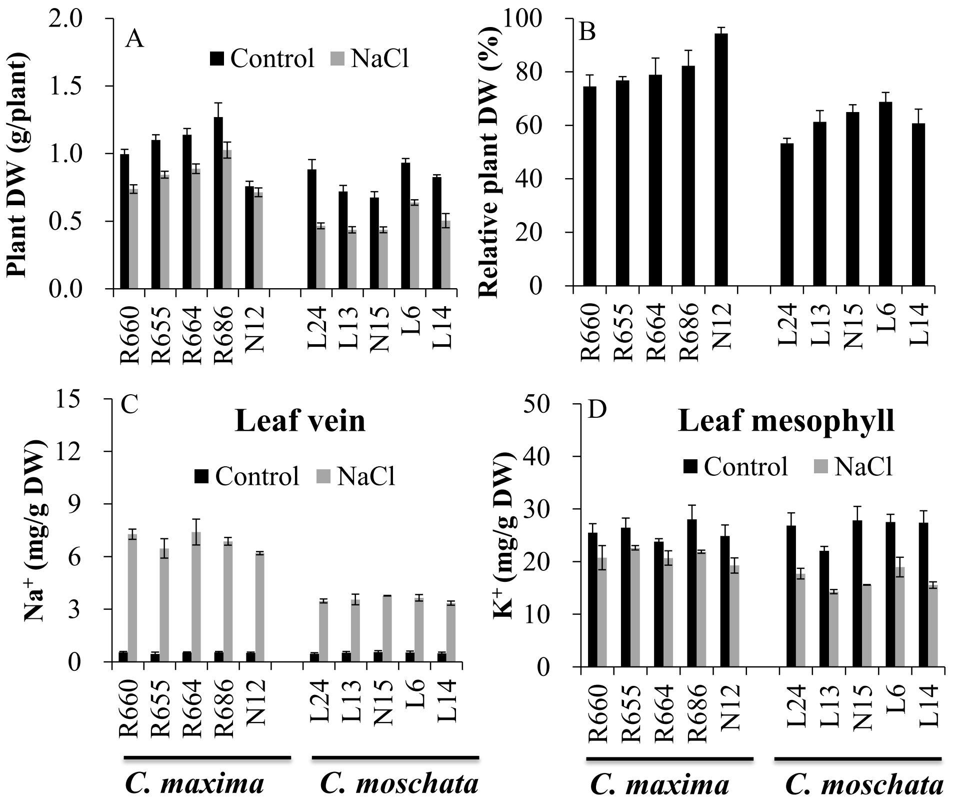

Fig. S2. Plant dry weight (DW, A), relative (% of control) plant DW (B), Na^{+} content in the leaf vein (C) and K^{+} content in the leaf mesophyll (D) of five *C. maxima* and five *C. moschata* genotypes grown hydroponically for 10 days under 100 mM NaCl stress. Values are the means ± SE (n=4).

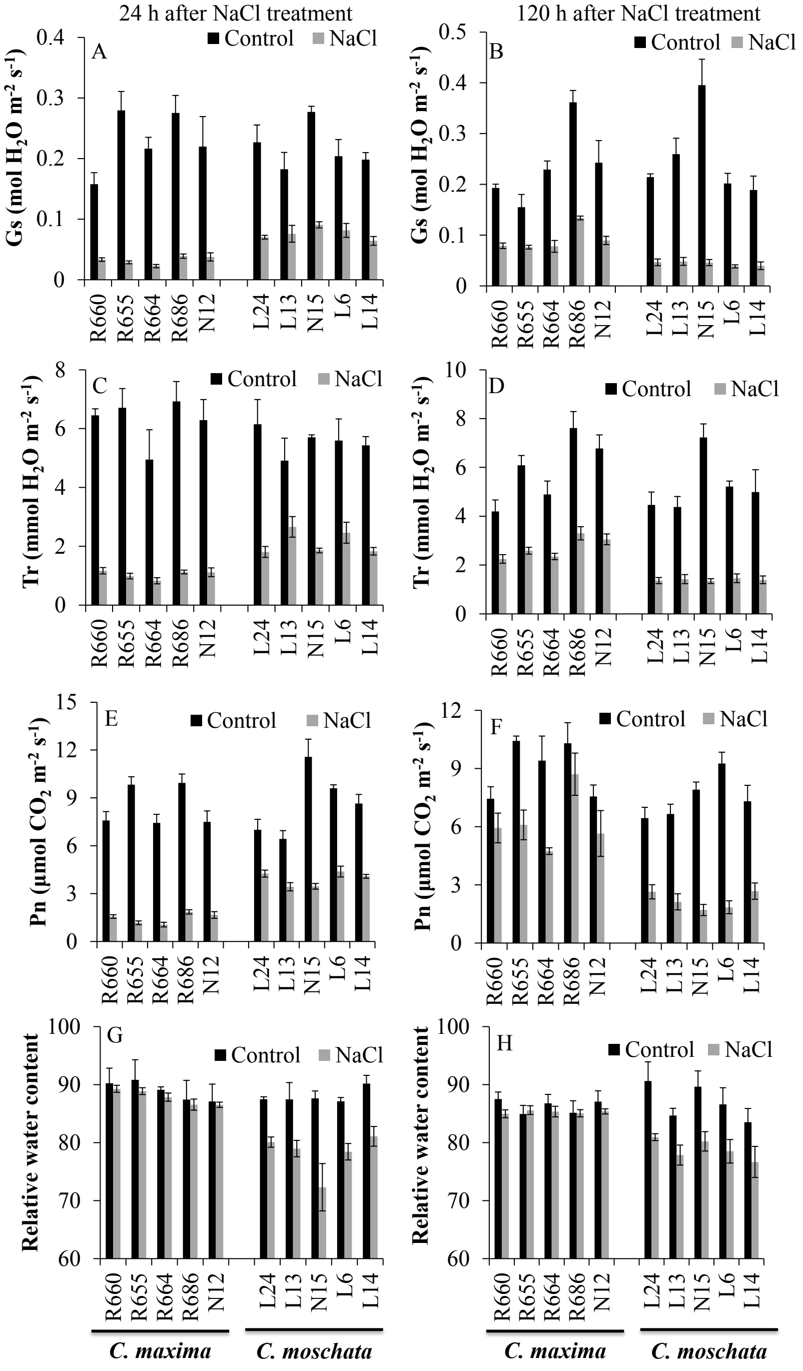

Fig. S3. Leaf gas stomatal conductance (Gs, A, B), transpiration rate (Tr, C, D), net photosynthetic rate (Pn, E, F), relative water content (G, H) of five *C. maxima* and five *C. moschata* genotypes grown hydroponically for 24 h or 120 h after 100 mM NaCl treatment. Values are the means ± SE (n=4).

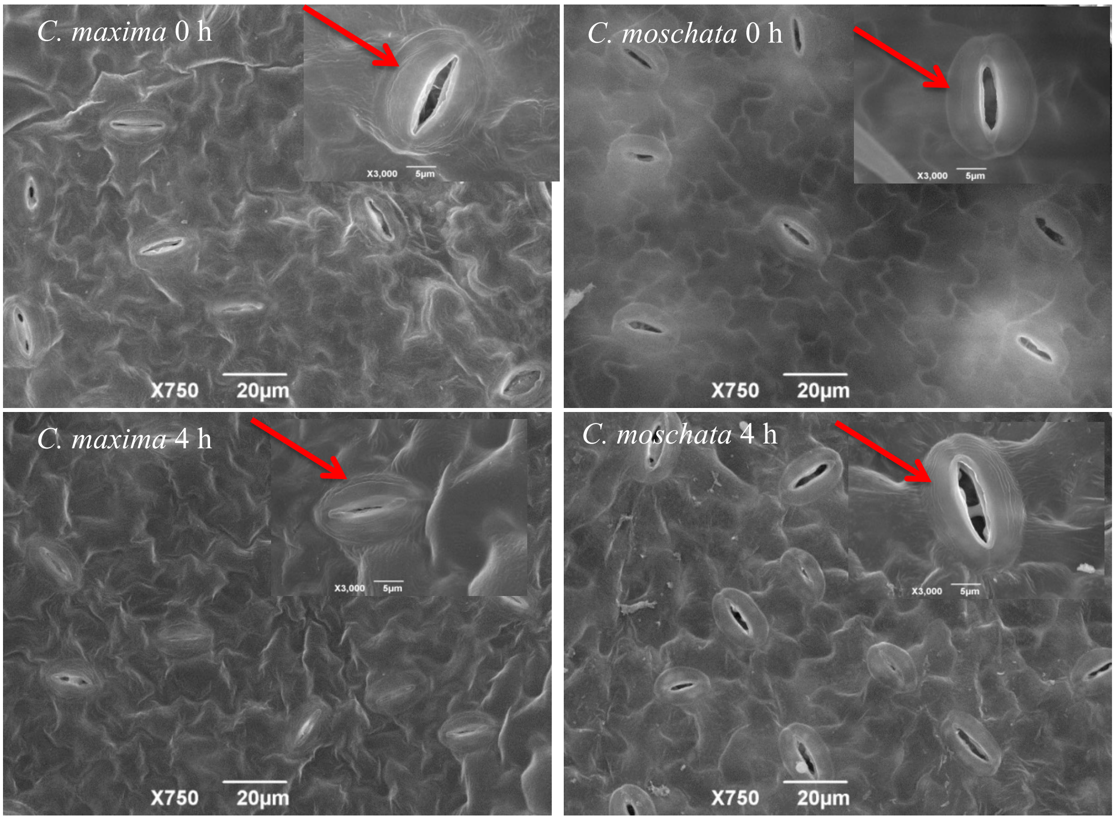

Fig. S4. Leaf stomatal aperture observation (red arrow indicates the magnified image of a stomata) of *C. maxima* (N12) and *C. moschata* (N15) grown hydroponically at 0 h and 4 h after 100 mM NaCl treatment.

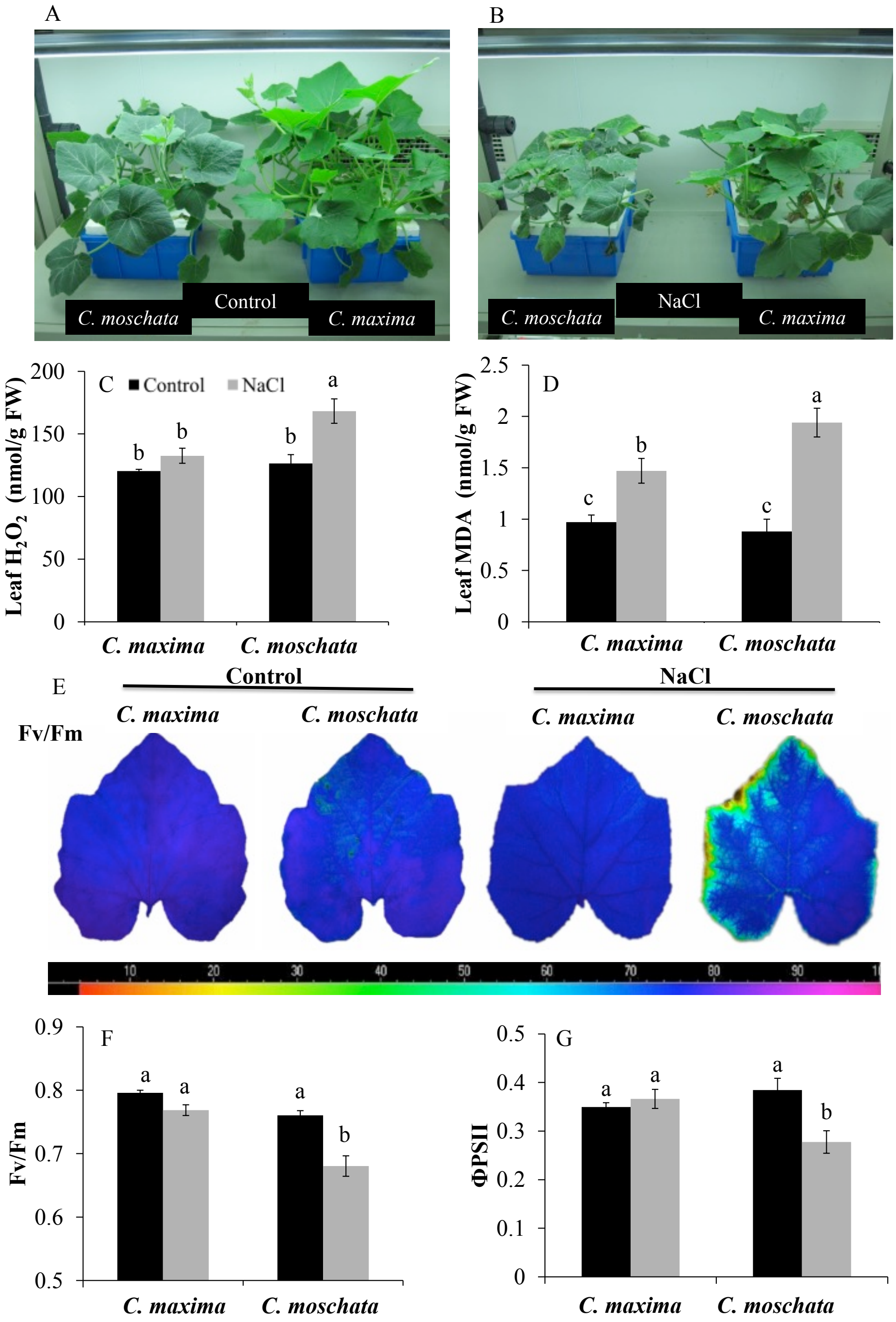

Fig. S5. Growth (A, B), concentration of leaf H_2O_2 (C) and MDA (malonaldehyde, D), Fv/Fm (the maximum quantum yield of PSII, E, F) and ΦPSII (quantum efficiency of electron transfer at PSII, G) of *C. maxima* (N12) and *C. moschata* (N15) grown hydroponically under Control (0 mM NaCl) and NaCl (100 mM) for 10 days. Different letters denote significantly ($P \leq 0.05$) different values according to Fisher's LSD test. Values are the means ± SE (n=3-5).

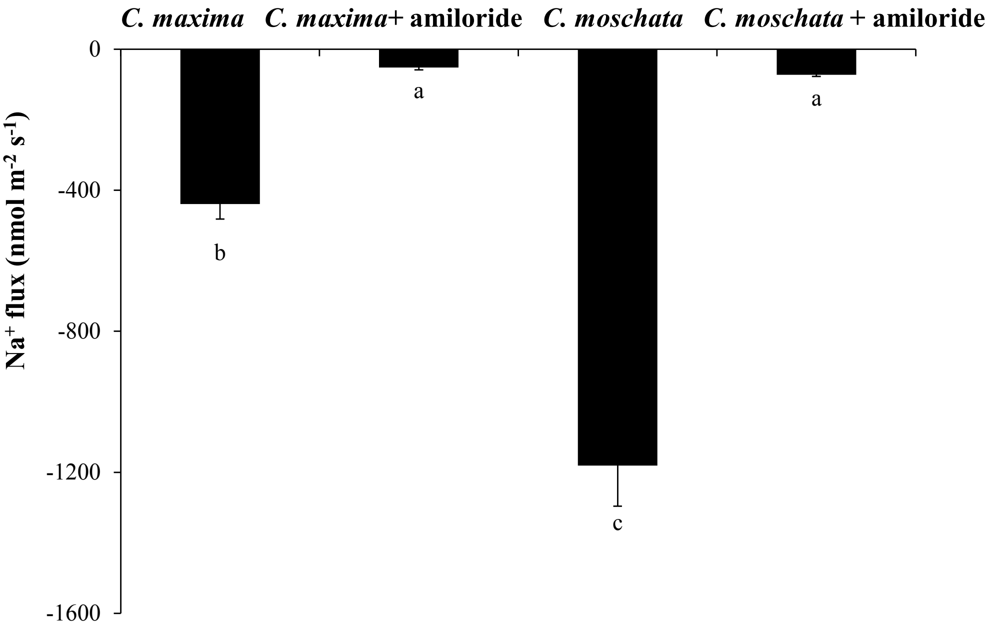

Fig. S6. Net Na^{+} flux from root epidermis of *C. maxima* (N12) and *C. moschata* (N15) after removal of NaCl, with or without 100 μM amiloride incubation. The plants were treated with 100 mM NaCl for 24 h before their transfer to Na-free solution. Different letters denote significantly ($P \leq 0.05$) different values according to Fisher's LSD test. The sign convention for the measurements is 'efflux negative'. Values are the means ± SE (n=6).

Supplementary Tables

Table S1. List of primer sequences used for qRT-PCR analysis

species	Genes	Forward Primer	Reversed Primer	Accession
C. maxima	*HKT1*	ACGTGCTTGAAAGCAAAGGC	CCGGATACAAACACCCTCCC	CmaCh10G003540
	NHX6	TTCTTCGCCGGCATAGAGTC	GTGAAAGTTGAACCACGCCC	CmaCh13G003240
	NHX4	CCGCACGGGACTATATTGCT	CTCCGAACACATGAAGGGCT	CmaCh17G011310
	KUP6	TTGCTTTGATTGGGGCTTGC	CAGCCCAGAAACAGCAGAGA	CmaCh11G014180
	KEA6	GTCACGATCGGCACCCTTAT	CAGAATTCCCACCCAGCACT	CmaCh12G006360
	NCED3	GAGACAGAACGGCTGGTTCA	CCATGGAGTTCCCCGATAGC	CmaCh07G001000
	EF1a	GCCTCAAACTCCAAGGATGA	GGCTCCTTCTCGAGTTCCTT	CmaCh08G010190
C. moschata	*HKT1*	CATCTATTGTCGGGGGTCCG	AATCCCACCGGAGTTGTTCC	CmoCh10G003830
	NHX6	GGGCGTGGTTCAACTTTCAC	AGCAAACGTCACAATTGCCC	CmoCh13G003420
	NHX4	CCGCACGGGACTATATTGCT	CTCCGAACACATGAAGGGCT	CmoCh01G011470
	KUP6	ACGAGTACGAGGCATTGGTC	GAAGGCCGGGAGGTTTGTTA	CmoCh11G017020
	KEA6	TGTTCTTCTCAGCCGTGCTT	TTGGACAAGGCTCGACAGAC	CmoCh12G005760
	NCED3	CGTGCCAAAACCTGAACCTG	AACCAGAGCATCCTCAACCG	CmoCh16G004950
	EF1a	GCCTCAAACTCCAAGGATGA	GGCTCCTTCTCGAGTTCCTT	CmoCh17G001050

All primers were designed based on a published mRNA of *C. maxima and C. moschata* on Cucurbit Genomics Database (http://cucurbitgenomics.org) using Primer 5 software. *EF1*a is the reference gene for qRT-PCR analysis.

Table S20. Significant differentially expressed (padj < 0.05, |log2FoldChange| > 1) genes in the leaf vein transcriptome of *C. maxima* and *C. moschata* compared with leaf mesophyll

ID	$Log_2FoldChange$	Annotation	Arabidopsis homologs
***C. maxima* under control (0 mM NaCl)**			
CmaCh10G003540	2.15	membrane Na^+ transporter	At4g10310/HKT1
CmaCh01G006810	1.12	potassium transporter	At2g30070/KUP1
CmaCh11G014180	-1.12	potassium transporter	At1g70300/KUP6
CmaCh15G003260	5.17	potassium transporter	At1g70300/KUP6
CmaCh10G007120	2.1	glutathione-regulated potassium-efflux system protein	At2g19600/KEA4
CmaCh18G006140	5.13	outward rectifying potassium channel protein	At5g55630/TPK1
CmaCh01G010790	1.1	outward rectifying potassium channel protein	At5g55630/TPK1
CmaCh18G004630	2.9	potassium channel	At5g46240/KAT1
CmaCh09G004740	2.2	potassium voltage-gated channel subfamily H member	At2g26650/AKT1
CmaCh04G005290	-1.58	potassium voltage-gated channel subfamily H member	At4g22200/AKT2
CmaCh06G009140	2.42	potassium voltage-gated channel subfamily H member	At3g02850/SKOR
CmaCh00G005050	2.38	potassium voltage-gated channel subfamily H member	At3g02850/SKOR
CmaCh10G010740	1.1	sodium/potassium/calcium exchanger	At1g08960/CCX5
CmaCh04G006430	1.7	9-cis-epoxycarotenoid dioxygenase	At3g14440/NCED3
CmaCh16G004600	2.08	9-cis-epoxycarotenoid dioxygenase	At3g14440/NCED3
***C. maxima* under NaCl (100 mM NaCl)**			
CmaCh10G003540	2.57	membrane Na^+ transporter	At4g10310/HKT1
CmaCh11G012510	-1.07	sodium/hydrogen exchanger	At3g05030/NHX2
CmaCh01G011010	1.42	sodium/hydrogen exchanger	At5g55470/NHX4
CmaCh01G006810	1.84	potassium transporter	At2g30070/KUP1
CmaCh15G003260	3.33	potassium transporter	At1g70300/KUP6
CmaCh04G017160	1.71	potassium transporter	At1g70300/KUP6
CmaCh10G007120	1.61	glutathione-regulated potassium-efflux system protein	At2g19600/KEA4
CmaCh18G006140	2.79	outward rectifying potassium channel protein	At5g55630/TPK1
CmaCh09G004740	2.16	potassium voltage-gated channel subfamily H member	At2g26650/AKT1
CmaCh04G005290	-1.99	potassium voltage-gated channel subfamily H member	At4g22200/AKT2
CmaCh18G004630	4.47	potassium channel	At5g46240/KAT1
CmaCh00G005050	1.8	potassium voltage-gated channel subfamily H member	At3g02850/SKOR
CmaCh06G009140	1.66	potassium voltage-gated channel subfamily H member	At3g02850/SKOR
CmaCh04G001110	-1.37	potassium transporter	At3g56290/uncharacterized
CmaCh07G001000	-1.66	9-cis-epoxycarotenoid dioxygenase	At3g14440/NCED3
***C. moschata* under control (0 mM NaCl)**			
CmoCh10G003830	3.9	membrane Na^+ transporter	At4g10310/HKT1
CmoCh17G011050	-1.61	sodium/hydrogen exchanger	At3g05030/NHX2
CmoCh01G007100	2.24	potassium transporter	At2g30070/KUP1
CmoCh15G003380	1.96	potassium transporter	At1g70300/KUP6
CmoCh08G010050	2.15	potassium voltage-gated channel subfamily H member	no homolog

C. moschata under NaCl (100 mM NaCl)

CmoCh10G003830	4.8	membrane Na^+ transporter	At4g10310/HKT1
CmoCh01G007100	1.7	potassium transporter	At2g30070/KUP1
CmoCh04G018020	1.46	potassium transporter	At1g70300/KUP6
CmoCh15G003380	1.43	potassium transporter	At1g70300/KUP6
CmoCh06G011200	-1.3	glutathione-regulated potassium-efflux system protein	At4g00630/KEA2
CmoCh04G024110	1.26	potassium voltage-gated channel subfamily H member	At3g02850/SKOR
CmoCh04G005660	-1.04	potassium channel	At4g22200/AKT2
CmoCh08G010050	2.58	potassium voltage-gated channel subfamily H member	no homolog
CmoCh14G013720	-1.57	outward-rectifying potassium channel 4-like protein	At1g52565/uncharacterized
CmoCh04G001080	-1.84	potassium transporter	At3g56290/uncharacterized
CmoCh03G013970	-1.9	9-cis-epoxycarotenoid dioxygenase	At3g14440/NCED3
